# An exploratory randomised double-blind and placebo-controlled phase 2 study of a combination of baclofen, naltrexone and sorbitol (PXT3003) in patients with Charcot-Marie-Tooth disease type 1A

**DOI:** 10.1186/s13023-014-0199-0

**Published:** 2014-12-18

**Authors:** Shahram Attarian, Jean-Michel Vallat, Laurent Magy, Benoît Funalot, Pierre-Marie Gonnaud, Arnaud Lacour, Yann Péréon, Odile Dubourg, Jean Pouget, Joëlle Micallef, Jérôme Franques, Marie-Noëlle Lefebvre, Karima Ghorab, Mahmoud Al-Moussawi, Vincent Tiffreau, Marguerite Preudhomme, Armelle Magot, Laurène Leclair-Visonneau, Tanya Stojkovic, Laura Bossi, Philippe Lehert, Walter Gilbert, Viviane Bertrand, Jonas Mandel, Aude Milet, Rodolphe Hajj, Lamia Boudiaf, Catherine Scart-Grès, Serguei Nabirotchkin, Mickael Guedj, Ilya Chumakov, Daniel Cohen

**Affiliations:** 1 Centre de référence des maladies neuromusculaires et de la SLA, Pôle des neurosciences Cliniques, AP-HM et Aix Marseille Université, Marseille, France; 2 CIC-Centre de Pharmacologie Clinique et D’Evaluations Therapeutiques, AP-HM et Aix Marseille Université, Marseille, France; 3 CHU de Limoges - Hôpital Dupuytren, 2 Avenue Martin Luther King, 87042 Limoges, France; 4 CHU Lyon Sud, 165 Chemin du Grand Revoyet, 69495 Lyon, France; 5 CHRU de Lille - Hôpital Roger Salengro, rue Emile Laine, 59037 Lille, France; 6 CHU de Nantes - Hôtel Dieu, 1 place Alexis Ricordeau, 44093 Nantes, France; 7 CHU de Paris - Groupe Hospitalier Pitié-Salpétrière, 47-83 boulevard de l’Hôpital, 75013 Paris, France; 8 Admissions, 75017 Paris, France; 9 Faculty of Medicine, The University of Melbourne, Grattan St, Melbourne, VIC 3010 Australia; 10 Faculty of Economics, UCL Mons, Louvain, Belgium; 11 Carl M. Loeb University Professor Emeritus, Harvard University, Cambridge, MA 02138 USA; 12 Pharnext, 11, rue des Peupliers, 92130 Issy-Les-Moulineaux, Paris, France

**Keywords:** Charcot-Marie-Tooth, CMT1A, Clinical trial, Phase 2, Repurposing, Combination therapy

## Abstract

**Background:**

Charcot-Marie-Tooth type 1A disease (CMT1A) is a rare orphan inherited neuropathy caused by an autosomal dominant duplication of a gene encoding for the structural myelin protein PMP22, which induces abnormal Schwann cell differentiation and dysmyelination, eventually leading to axonal suffering then loss and muscle wasting. We favour the idea that diseases can be more efficiently treated when targeting multiple disease-relevant pathways. In CMT1A patients, we therefore tested the potential of PXT3003, a low-dose combination of three already approved compounds (baclofen, naltrexone and sorbitol). Our study conceptually builds on preclinical experiments highlighting a pleiotropic mechanism of action that includes downregulation of *PMP22*. The primary objective was to assess safety and tolerability of PXT3003. The secondary objective aimed at an exploratory analysis of efficacy of PXT3003 in CMT1A, to be used for designing next clinical development stages (Phase 2b/3).

**Methods:**

80 adult patients with mild-to-moderate CMT1A received in double-blind for 1 year Placebo or one of the three increasing doses of PXT3003 tested, in four equal groups. Safety and tolerability were assessed with the incidence of related adverse events. Efficacy was assessed using the Charcot-Marie-Tooth Neuropathy Score (CMTNS) and the Overall Neuropathy Limitations Scale (ONLS) as main endpoints, as well as various clinical and electrophysiological outcomes.

**Results:**

This trial confirmed the safety and tolerability of PXT3003. The highest dose (HD) showed consistent evidence of improvement beyond stabilization. CMTNS and ONLS, with a significant improvement of respectively of 8% (0.4% - 16.2%) and 12.1% (2% - 23.2%) in the HD group *versus* the pool of all other groups, appear to be the most sensitive clinical endpoints to treatment despite their quasi-stability over one year under Placebo. Patients who did not deteriorate over one year were significantly more frequent in the HD group.

**Conclusions:**

These results confirm that PXT3003 deserves further investigation in adults and could greatly benefit CMT1A-diagnosed children, usually less affected than adults.

**Trial registration:**

EudraCT Number: 2010-023097-40. ClinicalTrials.gov Identifier: NCT01401257. The Committee for Orphan Medicinal Products issued in February 2014 a positive opinion on the application for orphan designation for PXT3003 (EMA/OD/193/13).

**Electronic supplementary material:**

The online version of this article (doi:10.1186/s13023-014-0199-0) contains supplementary material, which is available to authorized users.

## Background

Charcot-Marie-Tooth disease Type 1A (CMT1A) (OMIM:118220 and Orphanet:ORPHA101081) is a rare disease belonging to the group of inherited, progressive, chronic sensory and motor peripheral neuropathies referred to as Charcot-Marie-Tooth (CMT) disease or also as “Hereditary Motor and Sensory Neuropathy” (HMSN). Presently incurable, CMT is the most common inherited disorder of the vperipheral nervous system [1,2]. CMT1A accounts for 70 to 80% of CMT Type 1 patients and for 40 to 50% of all CMT patients [3-5]. Thus, CMT1A accounts for 50% of patients with CMT, with an estimated prevalence of 1 in 5,000 [1,2,6]. CMT1A is caused by an autosomal dominantly transmitted intrachromosomal 17p11.2 duplication harbouring *PMP22*, a gene encoding a myelin protein [7,8]. *PMP22* 1.5-fold overexpression induces abnormal Schwann cell differentiation, homogeneous and diffuse nerve conduction slowing, and dysmyelination, eventually leading to axonal loss and muscle wasting. A typical feature of CMT1A includes weakness of the foot and lower leg muscles which may result in foot drop and a high-stepped gait with frequent tripping or falls [3,4]. Foot deformities are also characteristic due to weakness of the small muscles in the feet, as well as “inverted champagne bottle” lower legs appearance due to the loss of muscle bulk. Later in the disease, weakness and muscle atrophy may occur in the hands, resulting in difficulty with fine motor skills. The severity of symptoms is quite variable in different patients and even among family members suffering from the disease.

There is currently no approved treatment available for CMT1A disease. In preclinical studies, ascorbic acid (AA) was shown to promote myelination *in vitro* and to decrease *PMP22* expression [9-11], and its mechanism of action in the murine peripheral nervous system has recently started to emerge [12]. Following these findings, six clinical trials assessing efficacy and tolerability of 1- or 2-year AA treatment have been published [13-18], but no beneficial clinical effects are reported in any of these trials. Considering the debilitating nature of the disease and the absence of specific therapy there remains a pressing unmet medical need for an efficacious and safe treatment for CMT1A.

We have favoured the idea that diseases can be more efficiently treated by targeting multiple pathways [19]. PXT3003 combines three drugs currently approved for other indications: (RS)-baclofen (a γ-aminobutyric acid [GABA]-B receptor agonist, used to treat spasticity), naltrexone hydrochloride (an opioid receptor antagonist, used to treat opiate and alcohol addiction) and D-sorbitol (a natural metabolite playing a role in the polyol pathway and prescribed for intestinal disorders). In preclinical experiments, the combination moderately lowers *PMP22* mRNA expression while it has been shown to improve impaired myelination and performances in CMT1A transgenic rats [20] (companion manuscript). Additional mechanisms of action of PXT3003 may exist since the known targets of its components are expressed not only in Schwann cells but also in peripheral neurons [21,22]. Moreover, PXT3003 is able to stimulate some axonal regeneration in acute nerve crush model assessed by the amplitude of Compound Muscle Action Potential (CMAP) (companion manuscript). As it is well known that preclinical and clinical therapeutic efficacy poorly correlate [23] and as individual drugs of PXT3003 combination have rather high safety profile, we decided to rapidly test it in CMT1A patients before studying thoroughly its precise mechanism of action in various models. In this one-year double-blind, randomised, placebo-controlled, dose-ranging Phase 2 study, we explore the potential of PXT3003 for the treatment of CMT1A as a proof of concept to decide on further investigations. The primary objective of the study is to assess the clinical and laboratory safety and tolerability of 3 doses of PXT3003 administered orally for 12 months to CMT1A patients. The evaluation of the efficacy of PXT3003 is the secondary objective, yet of a particular importance for future investigations on PXT3003.

## Methods

### Patients

Patients were recruited into this double-blind, randomised, placebo-controlled Phase 2 study at six hospital sites in France (Marseille, Lille, Limoges, Lyon, Nantes and Paris) from December 2010 to October 2011; the study ended in November 2012 (EudraCT Number: 2010-023097-40, ClinicalTrials.gov Identifier: NCT01401257). Inclusion criteria were: age (18–65 years), CMT1A diagnosis based on clinical examination and confirmation by genotyping (duplication in 17p11.2), weakness in at least foot dorsiflexion, and a Charcot Marie Tooth Neuropathy Score (CMTNS) ≤ 20, i.e. a mild to moderate disability. Key exclusion criteria included any neurological disease other than CMT1A, the use of unauthorized concomitant treatments (including but not limited to baclofen, naltrexone, sorbitol, ascorbic acid, opioids, levothyroxine, and potentially neurotoxic drugs), history of significant hematologic, kidney or liver disease, insulin-dependent diabetes or porphyria. Women of childbearing age were excluded if they were pregnant, breastfeeding, or not using adequate contraception. The protocol was approved by the local ethics committee and regulatory authority (*Agence Française de Securité Sanitaire des Produits de Santé*). The study was done in accordance with the Declaration of Helsinki and Good Clinical Practice (GCP), and all patients gave written informed consent before participation.

### Selection of doses

The doses tested in human were chosen based on extrapolation [24] from effective doses tested in CMT1A rats for an oral administration twice daily in accordance with the available pharmacokinetic data of the three individual compounds.

### Randomisation, masking and blinding

After a screening visit, eligible patients were randomly assigned in a 1:1:1:1 ratio to receive daily for one year Placebo, Low dose (LD = 0.6 mg baclofen, 0.07 mg naltrexone and 21 mg sorbitol), Intermediate dose (ID = 1.2 mg baclofen, 0.14 mg naltrexone and 42 mg sorbitol) or High dose (HD = 6 mg baclofen, 0.7 mg naltrexone and 210 mg sorbitol) of PXT3003 (Additional file 1: Table S1). A block randomisation scheme stratified by study centre was used to ensure a balance of treatment groups within each centre. Once the eligibility of the patient was confirmed by the investigator, patients were assigned a randomisation number according to a randomisation process using an interactive web randomisation system (IWRS). Study assignments were numbered according to a material randomisation list, separate from the subject randomisation list. Each patient’s assigned medication was determined by his/her randomisation number. Blinding was ensured and both patients and investigators were unaware of the treatment allocation.

Both PXT3003 and Placebo were provided in amber glass bottles containing 100 mL of clear solution, with the same appearance and taste. The three dosages above contain a quantity of sorbitol small enough to be counterbalanced by the acetate buffer and easily masked by the banana flavouring, not allowing to differentiate them (by sight or taste) from Placebo. Study drug was taken orally, using an adaptable graduated plastic pipette for medication dispensation, twice a day (morning and evening) during 12 months.

### Procedures

Potentially eligible subjects were evaluated at screening visits that included history, confirmation of CMT1A genotyping, physical examination including vital signs measurement (blood pressure, heart rate and weight), electrocardiogram (ECG), concomitant medications, recording of CMTNS and Overall Neuropathy Limitations Scale (ONLS), centralised laboratory examinations blood samples including Aspartate Aminotransferase (AST), Alanine Aminotransferase (ALT), gamma-GT, total, conjugated and un-conjugated bilirubinaemia, alkaline phosphatase, creatininaemia, complete blood count (CBC) and differential count, platelet count, sodium, potassium, chloride and blood glucose. Blood pregnancy test was obtained for women of childbearing age at screening and after 12 months of treatment. If subjects met eligibility criteria, they were randomised and enrolled in the study. An independent Data Monitoring Committee (DMC) was established to review all safety data at specified intervals throughout the study. All patient data related to the study were recorded on an electronic case report form (eCRF).

### Safety and tolerability

We monitored the safety of PXT3003 during the study up to 30 days after the last day of study drug administration, based on adverse events reports from patients and laboratory tests (biochemistry and haematology). All adverse events, whether or not considered by the investigator to be related to the study medication, were to be recorded in the patient’s medical records and in the eCRF. Serious adverse events were reported by the study investigators. A last follow-up visit consisting of a clinical examination, adverse events records, concomitant treatments and laboratory tests was done 30 days after the last day of treatment. The primary endpoint is the Incidence of related Adverse Events (including possibly and probably related AE) with moderate or severe intensity.

Compliance was measured after 1, 3, 6, 9 and 12 months by returned empty bottle count and volume measurement (full and opened bottles). In the present study, mean (s.d.) duration of exposure overall was 11.69 (1.53) months and was similar among the four groups, and mean (s.d.) compliance with study drug was 97.3 (9.41%). Patients with compliance below 80% were considered as low compliant.

### Pharmacokinetics

Blood samples for pharmacokinetic (PK) assessment of baclofen were drawn at randomisation (at peak only, 90 minutes after the first dose intake), 1, 6 and 12 months (at trough and peak, before and after 90 minutes of drug intake) or at the end of study drug administration in the case of patient’s discontinuation. Plasma concentration of baclofen was measured using a validated high-performance liquid chromatography coupled with mass spectrometry (LC-MS/MS) in a central laboratory. Baclofen plasma concentration at trough and at peak allowed us to check the compliance of the study drug and to show the kinetic of the drug according to the 3 doses.

However, because of the very low doses of naltrexone, for most of the patients the plasmatic concentration was found below the lower limit of quantification (LLOQ) of the validated method (30 pg/mL) even at peak. At 12 months, naltrexone could be dosed at peak for only 16 patients (11 patients in the HD group, 3 in the ID group and 2 in the LD group), and at trough for only 2 patients in the HD group, while baclofen concentration could testify that the patient had correctly taken PXT3003. Sorbitol was not dosed for technical reasons as it could not be detected under the conditions of the sampling.

### Electrophysiology

Nerve conduction studies were performed using standard techniques at skin temperature of 32°C. They included motor and sensory responses of median and ulnar nerves of the non-dominant upper limb.

For *motor parameters* (amplitude of Compound Muscle Action Potential (CMAP), Motor Conduction Velocity (MCV) and Distal Motor Latency (DML)), the median nerve was stimulated at the wrist and antecubital fossa, and responses were recorded over the *abductor pollicis brevis*. The ulnar nerve was stimulated at the wrist and below the elbow, and responses were recorded over the *abductor digiti minimi*.

For *sensory parameters* (amplitude of Sensory Nerve Action Potential (SNAP) and Sensitive Conduction Velocity (SCV)), we used an antidromic method and ring electrodes by stimulating the nerves at the wrist. For the median nerve, the active electrode was placed at the base of the second digit and the reference electrode between the fourth and the fifth metacarpals of the second digit. For the ulnar nerve, the active electrode was placed at the base of the fifth digit and the reference electrode between the fourth and the fifth metacarpals of the fifth digit.

### Efficacy

Two composite neurological scores were used to address efficacy in this study. The *Charcot-Marie-Tooth Neuropathy Score (*CMTNS*)* was proposed and validated by Shy *et al.* to provide a single and reliable measure of impairment in CMT [25,26]. It is a 36-point scale based on 9 items comprising 5 of impairment (sensory symptoms, pin sensibility, vibration, strength arms and legs), 2 of activity limitations (motor symptoms arms and legs) and 2 of electrophysiology (amplitudes of ulnar CMAP and SNAP). Higher scores indicate worsening function, and the score categorises disability as mild (0–10), moderate (11–20) and severe (21–36). The *Charcot-Marie-Tooth Examination Score* (CMTES) is a sub-score of the CMTNS comprising only the first 7 items, excluding the 2 electrophysiological items CMAP and SNAP.

Although CMTNS is an accepted measure of CMT severity, its sensitivity to change is still debated and it was agreed that some CMTNS components are not sensitive, mainly because of floor and ceiling effects [27]. For instance, if the patient had surgery (fixation of the ankle), the ‘motor legs symptoms’ definitively scores 3, and no further decrease can be scored. The usefulness of the SNAP component is also limited as it is frequently absent in CMT1A patients who then receive the maximum score on their entry visit and thus have a low sensitivity to change. Komyathy *et al.* (2013) pointed out that sensory and motor symptoms items are based on subjective opinion from patients, concerning their leg and arm strength and loss of sensation in their legs, and that pin sensibility, vibration and strength are based on a neurological examination which depends on patient cooperation and examiner consistency to obtain reproducible results [28]. Finally, some measurement of CMTNS, such as SNAP or vibratory sensation, may decrease with age, so that scores from patients could increase slightly as they get older, independently of CMT. A second, modified version of the scale (CMTNS2) was therefore proposed by Murphy *et al.* (2011), at a time when the present study was ongoing, to attempt to reduce floor and ceiling effects and to standardise patient assessment [29]. Although CMTNS sensitivity in detecting the effects of a therapeutic intervention has not been demonstrated up to now, it is the only CMT-specific outcome measure (although not specific to CMT1A subtype), used as primary outcome in the clinical trials for ascorbic acid [16-18], and therefore remains of particular interest for inter-study comparisons.

The *Overall Neuropathy Limitations Scale* (ONLS) was derived and improved from the ODSS by Graham and Hughes (2006) to measure limitations in the everyday activities of the upper limbs (rated on 5 points) and the lower limbs (rated on 7 points) [30]. The total score goes from 0 (= no disability) to 12 (= maximum disability).

Although the functioning of patients with peripheral neuropathy may be influenced by other factors in addition to their physical capacity, ONLS measures the perceived ability of the patient to move and enjoy a normal life, and thus is expected to be associated with quality of life. It was initially validated in a pool of 100 patients with diverse peripheral neuropathies (mainly of dysimmune origin but including 9 CMT patients), and it showed significant relationships with measures of impairment, disability and quality of life, although it did not correlate significantly with the SF-36 Role Limitation Physical Subscale in patients with neuropathies other than Guillain-Barré Syndrome (GBS) and Chronic Inflammatory Demyelinating Polyneuropathy (CIDP). ONLS was recommended as a core disability scale for CMT1A studies [26,27]. Its reliability was further validated in studies of CMT patients [31] and it was also used in ascorbic acid therapeutic trials on CMT1A patients [16,17].

The importance of both scores to assess impairment and disability in CMT1A led us to consider them *a priori* as main efficacy outcomes.

All other individual efficacy outcome measurements were considered as secondary endpoints and were treated equally. They included:

- *i) Functional measures:*
  - 6-Minute Walk Test (6MWT) [32] to assess gait velocity by the distance walked during six minutes (in metres).
  - 9-Hole Peg Test (9HPT) to assess finger dexterity by the time required to put nine pegs in nine holes (in seconds, non-dominant hand considered).
  - Quantified Muscular Testing (QMT) [33] to assess muscle strength by Ankle Dorsiflexion (in Newton metres, mean of left and right side considered) and Hand Grip (in kilogrammes, non-dominant hand considered).
- *ii) Electrophysiological parameters:*
  - Amplitude of Compound Muscle Action Potential (CMAP, in millivolt), Motor Conduction Velocity (MCV, in metres per second) and Distal Motor Latency (DML, in millisecond) measured from the mean motor responses of the median and ulnar nerves (non-dominant side considered).
  - Amplitude of Sensory Nerve Action Potential (SNAP, in microvolt) and Sensitive Conduction Velocity (SCV, in metres per second) measured from the mean sensory responses of the median and ulnar nerves (non-dominant side considered). The nerve sensory measures have to be interpreted with caution because of a particularly high content of missing values in SCV and of 0 values in SNAP, the latter reflecting measures below the detection threshold.
  Myelin state of non-degenerated axons is generally assessed by MCV, DML and SCV while CMAP and SNAP inform on the degree of axonal loss, but it must be stressed that the variability attached to these measures is high.
- *iii) Self-assessment-based measurements:*
  - Visual Analog Scales (VAS) for pain, fatigue and global state were assessed by the patient;
  - Clinical Global Impression (CGI) for global improvement, illness severity and therapeutic effect was assessed by the physician.

All efficacy outcomes were assessed at randomisation and at 6 and 12 months.

### Additional exploratory outcomes

A set of additional, exploratory secondary outcomes were monitored during the study.

- Intra-epidermal axon density in cutaneous biopsy of the lateral malleolus.
- mRNA expression of *PMP22* in cutaneous biopsy of the lateral malleolus.
- Plasma concentrations of a series of bio-chemical biomarkers.

Their analysis and interpretation of the results are still ongoing, and will be the subject of future publications.

### Between-sites standardisation

To standardise the outcome measures, the sponsor supplied the material required for the 6MWT in the hospital ward, the required tool to perform the 9HPT and the required tool (MicroFet2 Jamar) to perform QMT. A technical document detailed the techniques and material used for these evaluations. Before the study began, all examiners attended training sessions; an additional training session was implemented at the opening of each study site; furthermore, an audit was conducted during the study on each site to confirm that instructions were correctly followed. Moreover, all analyses (pharmacokinetic and clinical laboratory evaluations) were centralised and performed in two central laboratories.

### Statistical analysis

#### Analysis population

All the analyses were conducted on the Full Analysis Set (all randomised patients, on an intent-to-treat basis).

#### Baseline analysis

Demographic and clinical patient characteristics were presented for each group [34].

#### Safety and tolerability analysis

Safety and tolerability analyses were based on the reported treatment-emergent, adverse events (TEAEs) and other safety information (vital signs, electrocardiogram and laboratory tests). The percentage of patients with treatment-emergent adverse events after one year was descriptively compared between groups and tested by a Fisher’s Exact Test.

#### Efficacy analysis

The composite scores used in this study (CMTNS and ONLS) were considered as the main efficacy outcomes. The monotonicity of the dose-effect was tested through a Spearman’s rank correlation between percentage of improvement and doses (numerically coded as Placebo = 0, LD = 1, ID = 2, HD = 3). Differences between treatment groups were assessed by Analysis of Covariance (ANCOVA) on log-transformed values by adjusting for baseline values. Estimates (LS-means) were provided as mean percentage change over baseline. This analysis was performed independently on CMTNS and ONLS. The significance of the treatment effect on both CMTNS and ONLS was then assessed through the significance of the O’Brien’s OLS test [35,36] considered as the most powerful method for the statistical inference of multiple outcomes. In the context of the assessment of a combination of old drugs restrained to very low doses, we suspected that the low dose and middle dose are *a priori* located below the inflexion point of the expected logistic dose–response function. Consequently, the Minimum Effective Dose search was performed by closed Step-Down procedure for comparing ordered doses with a control on Helmert contrasts (SD2H algorithm, the i*th* dose level mean is compared to the average of all the lower dose level means, including the zero dose level) [37,38]. Based on the assumption of monotonicity of the doses on the main endpoints, the detection of a Minimum Effective Dose among groups and a linear correlation coefficient of at least *R* = 0.75 between baseline and final values, a Standardised Mean Difference (also known as Cohen’s *d*) of at least s.m.d. = 0.5 should be detected with a power of at least 80% when the sample size reaches 20 patients per group and at a one-tailed 5% significance level [39,40]. The treatment-by-centre interaction was assessed on CMTNS and ONLS and we concluded that there was no interaction. The ANCOVA analysis was repeated for the other efficacy outcomes (6MWT, 9HPT, Ankle Dorsiflexion, Grip, CMAP, MCV, DML, SNAP, SCV, VAS and CGI). Finally, we considered the patients non-deteriorated (including stabilization or improvement) after the 12-month treatment. Deterioration was evaluated by the percentage of change averaged over CMTNS and ONLS. The proportions of non-deteriorated patients in each group were compared using a logistic regression model [41].

#### General procedures

Statistical analyses were performed with *R* version 3.0.1 (http://cran.r-project.org). Data distribution and within-group variation were preliminary assessed in order to guide our methodological choices [39-41]. Missing data imputation was performed by mixed model approach considered as the most appropriate technique for intent to treat longitudinal studies [42]. Statistical tests were conducted at a 5% significance level.

## Results

**Figure 1:**
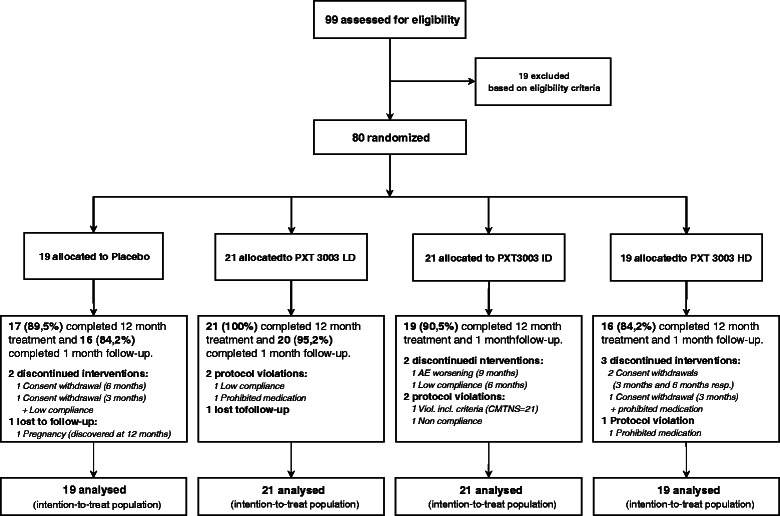
**Diagram of the study design.** The number of CMT1A patients randomised to each group and reasons for withdrawal are indicated.

Only seven patients (8.7%) left the study before completion. Demographic features, clinical characteristics and reasons for drop-out were comparable between the groups (Table 1 and Figure 1). Pharmacokinetic analyses assessed drug exposure and compliance (Additional file 2: Table S2).

**Table 1:** **Characteristics at baseline (Full Analysis Set,** ***n*** **= 80)**

	**Placebo** **(** ***n*** **= 19)**	**PXT3003 LD** **(** ***n*** **= 21)**	**PXT3003 ID** **(** ***n*** **= 21)**	**PXT3003 HD** **(** ***n*** **= 19)**
**Mild/Moderate**	3 (16%)/16 (84%)	4 (19%)/17(81%)	5 (24%)/16 (76%)	3 (16%)/16 (84%)
**Women/Men**	11 (58%)/8 (42%)	14 (67%)/7 (33%)	13 (62%)/8 (38%)	10 (53%)/9 (47%)
**Age (years)**	43.2 (12.2)	47.9 (14.9)	44.3 (12.7)	44.6 (11.2)
**Body Mass Index**	25.0 (3.9)	24.5 (4.0)	23.5 (3.5)	23.6 (4.6)
**Disease Duration (years)**	7.1 (5.5)	10.2 (13.1)	6.6 (4.5)	8.9 (5.5)
**CMTNS**	14.3 (3.8)	14.2 (4.1)	13.0 (4.0)	13.8 (3.4)
**ONLS**	3.1 (1.1)	3.3 (1.0)	3.5 (0.9)	3.6 (0.8)
**6MWT (m)**	468.2 (99.9)	473.1 (70.9)	450.7 (71.1)	429.3 (83.7)
**9HPT (s)**	17.2 (2.5)	16.1 (3.9)	18.4 (4.7)	20.8 (7.8)
**Ankle Dorsiflexion (Nm)**	7.8 (6.6)	9.1 (5.0)	8.3 (5.6)	8.2 (6.1)
**Grip (kg)**	22.6 (10.7)	21.6 (6.1)	23.1 (9.2)	20.6 (10.4)
**CMAP (milliV)**	3.7 (2.0)	4.0 (1.8)	3.7 (2.1)	3.4 (2.3)
**MCV (m/s)**	21.5 (3.6)	22.7 (4.7)	20.8 (4.8)	20.5 (5.3)
**DML (ms)**	8.6 (2.2)	7.9 (2.1)	8.2 (1.8)	8.2 (1.9)
**SNAP (microV)**	2.6 (3.2)	2.3 (3.0)	2.6 (3.8)	2.2 (2.7)
**SCV (m/s)**	31.1 (14.8)	29.4 (8.2)	31.3 (9.4)	29.9 (7.7)

Data are count (%) or mean (s.d.). CMTNS = Charcot-Marie-Tooth Neuropathy Score; ONLS = Overall Neuropathy Limitations Scale; 6MWT = 6-Minute Walk Test; 9HPT = 9-Hole Peg Test; CMAP = Amplitudes of Compound Muscle Action Potentials; MCV = Motor Conduction Velocity; DML = Distal Motor Latency; SNAP = Amplitudes of Sensory Nerve Action Potentials; SCV = Sensitive Conduction Velocity. Mild: CMTNS ≤ 10; Moderate: 11 ≤ CMTNS ≤ 20.

### Safety and tolerability

Safety and tolerability of PXT3003 were good, confirming results from our regulatory preclinical studies and the anticipated absence of toxicity at the chosen low doses. Intake of the treatment did not indicate any influence on the results of vital signs (blood pressure, heart rate and weight), electrocardiogram measurements and laboratory tests (biochemistry and haematology). The percentage of patients with treatment-emergent adverse events (TEAEs) after one year was similar across treatment groups (47% in Placebo, 23% in LD, 33% in ID, 31% in HD, *P* = 0.48). Most of the related TEAEs were mild and benign (Table 2).

**Table 2:** **Most Frequent Study, Medication-Related, Treatment-Emergent Adverse Events (Frequency > 2.0% of the total patients) by MedDRA System Organ Class and Preferred Term (Full Analysis Set,** ***n*** **= 80)**

	**PXT3003**						**Placebo**		**Total**	
	**High dose**		**Intermediate dose**		**Low dose**					
	**(** ***n*** **= 19)**		**(** ***n*** **= 21)**		**(** ***n*** **= 21)**		**(** ***n*** **= 19)**		**(** ***n*** **= 80)**	
	***n***	**(%)**	***n***	**(%)**	***n***	**(%)**	***n***	**(%)**	***n***	**(%)**
**Any adverse event**	6	31.6	7	33.3	5	23.8	9	47.4	27	33.8
**Gastrointestinal disorders**	4	21.1	1	4.8	1	4.8	6	31.6	12	15.0
**Nausea**	2	10.5	1	4.8	0	0.0	3	15.8	6	7.5
**Abdominal pain**	2	10.5	0	0.0	0	0.0	1	5.3	3	3.8
**Diarrhoea**	1	5.3	0	0.0	0	0.0	1	5.3	2	2.5
**Nervous system disorders**	3	15.8	2	9.5	2	9.5	2	10.5	9	11.3
**Dizziness**	1	5.3	0	0.0	2	9.5	2	10.5	5	6.3
**Headache**	0	0.0	2	9.5	0	0.0	0	0.0	2	2.5
**Somnolence**	2	10.5	0	0.0	0	0.0	0	0.0	2	2.5
**General disorders and administration site conditions**	0	0.0	1	4.8	1	4.8	3	15.8	5	6.3
**Fatigue**	0	0.0	1	4.8	1	4.8	1	5.3	3	3.8
**Musculoskeletal and connective tissue disorders**	1	5.3	1	4.8	1	4.8	1	5.3	4	5.0
**Muscle spasms**	1	5.3	1	4.8	0	0.0	0	0.0	2	2.5
**Renal and urinary disorders**	1	5.3	0	0.0	0	0.0	1	5.3	2	2.5

MedDRA = Medical Dictionary for Regulatory Activities. Study medication relationship was assumed if the relationship to study medication was judged as ‘possible’ or ‘not assessable’ by the investigator or if the judgement was missing.

### Efficacy

**Figure 2:**
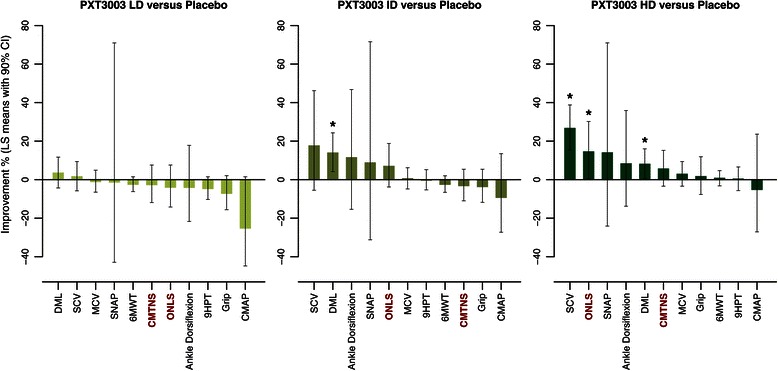
**Summary of the efficacy analysis, active doses versus Placebo.** Least squares mean percentages of relative improvement after 12 months along with 90% CI in all efficacy outcomes after 12 months for LD, ID and HD groups *versus* Placebo (obtained from Table 3). These estimates and CI were assessed by the ANCOVA efficacy analysis and sorted from highest to lowest value. A negative value of improvement means that the outcome was deteriorated after 12 months. Sample sizes: Placebo (*n =* 19), LD (*n =* 21), ID (*n =* 21), HD (*n =* 19). The two main efficacy outcomes, CMTNS and ONLS, are indicated with red bold characters. *P < 0.05.

**Figure 3:**
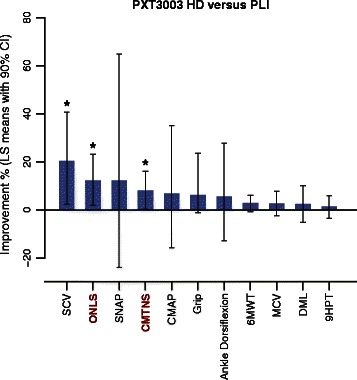
**Summary of the efficacy analysis, HD versus PLI.** Least squares mean percentages of relative improvement after 12 months with 90% CI in all efficacy outcomes after 12 months for HD *versus* PLI (obtained from Table 4), assessed by the ANCOVA efficacy analysis and sorted from highest to lowest value. Sample sizes: PLI (*n =* 61), HD (*n =* 19). The two main efficacy outcomes, CMTNS and ONLS, are indicated with red bold characters. *P < 0.05.

Efficacy data suggested an increasing dose-effect for PXT3003, significant on ONLS, DML and SCV (Table 3 and Figure 2). For most of the efficacy outcomes, the best improvement was observed in the HD group (CMTNS, ONLS, 6MWT, 9HPT, Grip, MA, MCV and SCV). A Step-Down search of the Minimum Effective Dose identified the High dose as the most promising dose (Additional file 3: Table S3). The other dose groups (LD, ID) can therefore be assimilated to Placebo, and the pool of the Placebo, LD and ID groups constitute from now the pooled control group termed PLI (Table 4 and Figure 3).

**Table 3:** **Response to PXT3003 on efficacy outcomes in treatment groups, with comparisons of active doses** ***versus*** **Placebo (Full Analysis Set,** ***n*** **= 80)**

	**Mean % of improvement**				**PXT3003 LD** ***versus*** **Placebo**		**PXT3003 ID** ***versus*** **Placebo**		**PXT3003 HD** ***versus*** **Placebo**		**Dose-effect**	
	**Placebo** **(** ***n*** **= 19)**	**PXT3003 LD** **(** ***n*** **= 21)**	**PXT3003 ID** **(** ***n*** **= 21)**	**PXT3003 HD** **(** ***n*** **= 19)**	**Estimate**	***P*** **-value**	**Estimate**	***P*** **-value**	**Estimate**	***P*** **-value**	**Correlation**	***P*** **-value**
**CMTNS**	2.6 (17.5)	0.5 (23.2)	−3.1 (16.0)	**7.7 (18.4)**	−2.6 (−11.9;7.6)	0.67	−3.1 (−11.0;5.4)	0.74	5.5 (−3.4;15.2)	0.16	0.54	0.30
**ONLS**	−5.3 (19.3)	−6.5 (18.5)	4.8 (24.2)	**12.3 (28.4)**	−3.9 (−14.2;7.6)	0.72	6.9 (−3.8;18.8)	0.15	14.4 (0.55;30.2)	0.043*	0.28	0.006*
**6MWT (m)**	9.0 (8.3)	6.2 (8.3)	6.4 (9.4)	**9.9 (6.9)**	−2.4 (−6.2;1.5)	0.85	−2.4 (−6.6;2.0)	0.82	0.7 (−3.2;4.7)	0.38	0.11	0.16
**9HPT (s)**	4.9 (11.4)	−1.2 (11.7)	5.6 (9.9)	**7.8 (12.1)**	−4.6 (−10.3;1.5)	0.89	−0.2 (−5.3;5.2)	0.52	0.3 (−5.7;6.6)	0.47	0.15	0.092
**Ankle Dorsiflexion (Nm)**	20.2 (88.4)	−3.6 (43.0)	**81.5 (369.6)**	20.4 (64.1)	−4.0 (−21.7;17.8)	0.63	11.4 (−15.4;46.8)	0.26	8.2 (−13.8;35.9)	0.28	0.11	0.16
**Grip (kg)**	9.9 (24.2)	1.3 (15.6)	4.7 (12.5)	**11.7 (18.1)**	−7.1 (−15.6;2.1)	0.90	−3.6 (−11.8;5.4)	0.75	1.6 (−7.7;11.9)	0.39	0.12	0.15
**CMAP (milliV)**	34.4 (62.0)	1.4 (38.7)	22.9 (62.6)	**64.2 (208.5)**	−25.1 (−44.8;1.5)	0.94	−9.2 (−27.3;13.5)	0.77	−5.1 (−27.1;23.6)	0.63	−0.001	0.50
**MCV (m/s)**	3.7 (8.5)	3.0 (11.5)	5.7 (12.3)	**9.0 (17.6)**	−1.0 (−6.5;4.9)	0.61	0.5 (−4.8;6.2)	0.44	2.8 (−3.4;9.4)	0.23	0.11	0.18
**DML (ms)**	0.4 (8.8)	3.6 (21.7)	**15.3 (35.8)**	8.4 (21.7)	3.4 (−4.3;11.7)	0.24	13.8 (4.2;24.3)	0.009*	8.0 (0.59;16.0)	0.038*	0.21	0.035*
**SNAP (microV)**	12.4 (121.7)	11.5 (88.2)	**23.3 (128.4)**	5.2 (69.0)	−1.2 (−42.9;71.0)	0.52	8.7 (−31.2;71.6)	0.38	13.9 (−24.1;71.0)	0.29	0.09	0.30
**SCV (m/s)**	3.4 (11.0)	5.3 (11.2)	29.5 (63.4)	**30.5 (10.0)**	1.5 (−5.8;9.4)	0.36	17.5 (−5.5;46.2)	0.11†	26.6 (15.5;38.8)	0.00037*	0.42	0.01*

Data are mean % (s.d.) of improvement for each treatment group. Differences between treatment groups were assessed by Analysis of Covariance (ANCOVA) on log-transformed values by adjusting for baseline values. Estimates were provided as mean percentage change over baseline (90% CI). Dose-effect was tested through Spearman’s rank correlation. *P*-values are one-tailed. *P < 0.05; Boldface = best improvement within dosages. CMTNS = Charcot-Marie-Tooth Neuropathy Score; ONLS = Overall Neuropathy Limitations Scale; 6MWT = 6-Minute Walk Test; 9HPT = 9-Hole Peg Test; CMAP = Amplitudes of Compound Muscle Action Potentials; MCV = Motor Conduction Velocity; DML = Distal Motor Latency; SNAP = Amplitudes of Sensory Nerve Action Potentials; SCV = Sensitive Conduction Velocity.

**Table 4:** **Response to PXT3003 on efficacy outcomes in HD and in PLI, with comparisons of HD** ***versus*** **PLI (Full Analysis Set,** ***n*** **= 80)**

	**Mean % of improvement**		**PXT3003 HD** ***versus*** **PLI**	
	**PLI** **(** ***n*** **= 61)**	**PXT3003 HD** **(** ***n*** **= 19)**	**Estimate**	***P*** **-value**
**CMTNS**	−0.1 (19.0)	**7.7 (18.4)**	8.0 (0.4;16.2)	0.042*
**ONLS**	−2.2 (21.2)	**12.3 (28.4)**	12.1 (2.0;23.2)	0.024*
**6MWT (m)**	7.1 (8.6)	**9.9 (6.9)**	2.6 (−0.73;6.1)	0.099
**9HPT (s)**	3.1 (11.3)	**7.8 (12.1)**	1.2 (−3.4;6.0)	0.33
**Ankle Dorsiflexion (Nm)**	**33.1 (223.2)**	20.4 (64.1)	5.5 (−12.8;27.7)	0.32
**Grip (kg)**	5.1 (17.9)	**11.7 (18.1)**	6.0 (−1.2;13.7)	0.088
**CMAP (milliV)**	19.6 (56.5)	**64.2 (208.5)**	6.6 (−15.8;35.1)	0.33
**MCV (m/s)**	4.2 (10.9)	**9.0 (17.6)**	2.5 (−2.4;7.7)	0.21
**DML (ms)**	6.7 (25.6)	**8.4 (21.7)**	2.2 (−5.1;10.0)	0.31
**SNAP (microV)**	**15.9 (110.2)**	5.2 (69.0)	12.0 (−23.9;64.9)	0.31
**SCV (m/s)**	12.7 (38.0)	**30.5 (10.0)**	20.1 (2.4;40.8)	0.030*

Data are mean % (s.d.) of improvement for HD and for PLI after 12 months. Differences between treatment groups were assessed by Analysis of Covariance (ANCOVA) on log-transformed values by adjusting for baseline values. Estimates were provided as mean percentage change over baseline (90% CI). *P*-values are one-tailed. *P < 0.05; Boldface = best improvement within groups. CMTNS = Charcot-Marie-Tooth Neuropathy Score; ONLS = Overall Neuropathy Limitations Scale; 6MWT = 6-Minute Walk Test; 9HPT = 9-Hole Peg Test; CMAP = Amplitudes of Compound Muscle Action Potentials; MCV = Motor Conduction Velocity; DML = Distal Motor Latency; SNAP = Amplitudes of Sensory Nerve Action Potentials; SCV = Sensitive Conduction Velocity.

**Figure 4:**
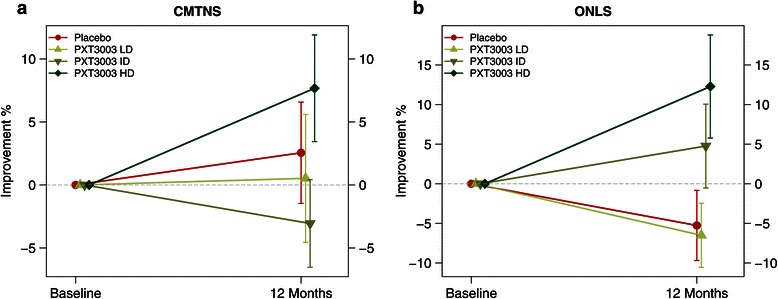
**Response to PXT3003 on clinical scales (Full Analysis Set,** ***n*** **= 80).** Mean % (s.e.m.) of improvement from baseline per group at 12 months for CMTNS **(a)** and ONLS **(b)**. Sample sizes: Placebo (*n* = 19), LD (*n* = 21), ID (*n* = 21), HD (*n* = 19).

CMTNS (Figure 4a) improved in the HD group compared to Placebo, and improved significantly when compared to PLI (8% of improvement, *P* = 0.042). ONLS (Figure 4b) improved significantly in the HD group compared to both Placebo (14.4% improvement, *P* = 0.043) and PLI (12.1% improvement, *P* = 0.024). The significance of the treatment effect on CMTNS and ONLS, assessed through the multiple endpoints O’Brien’s OLS test, confirmed the improvement of the HD group compared to Placebo (*P* = 0.036) and PLI (*P* = 0.0049). Among the functional measures (including 6MWT, 9HPT, Ankle Dorsiflexion and Hand Grip), 6MWT and Hand Grip showed a trend toward improvement in the HD group compared to PLI despite evidence of some training effect assessed from the Placebo group and PLI. The global significance of the treatment effect on the four functional measures taken together with O’Brien OLS test also confirmed an improvement in the HD group compared to PLI (*P* = 0.051).

Electrophysiological results suggested that myelin function might have been improved. DML was significantly decreased in the HD group when compared to Placebo (8%, *P* = 0.038), and SCV was significantly increased in HD when compared to Placebo (26.6%, *P* = 3.70×10^−4^) and PLI (20.1%, *P* = 0.03). MCV was increased in the HD group when compared to Placebo without reaching statistical significance but a trend to dose effect was observed (*P* = 0.18). This set of observations is consistent with the putative mechanisms of action of PXT3003 that may impact myelination, as shown *ex vivo* and *in vivo* with the CMT1A rat model. As expected, the rate of missing values in SCV was rather high (55%), as well as the proportion of 0 values in SNAP (39.8%).

The quality of life assessed by VAS and CGI measurements did not change when compared to Placebo (Additional file 4: Table S4). In CMT, such self-assessment-based scores are known to be independent with respect to disease severity, and their ability to reflect a therapeutic effect in this short term treatment cannot be confirmed [43,44].

### Patients non-deteriorating after one year under HD appear less affected at baseline

The proportion of patients non-deteriorated after 12 months was similar in the Placebo, LD and ID groups (around 48%), and significantly higher in the HD group (79%, Relative Risk 1.66, *P* = 0.01, Table 5). Comparison of baseline characteristics between non-deteriorated and deteriorated patients treated with PXT3003 HD suggested that non-deteriorated patients were less severely affected by the disease (Additional file 5: Table S5a): the baseline value for most outcomes appeared better in non-deteriorated patients, significantly for MCV and DML (*P* = 0.0051 and 0.01 respectively). The same comparison was performed in the Placebo group as a negative control and showed no trend or significant differences (Additional file 5: Table S5b).

**Table 5:** **Proportion of non-deteriorated patients (Full Analysis Set,** ***n*** **= 80)**

	**Placebo** **(** ***n*** **= 19)**	**PXT3003 LD** **(** ***n*** **= 21)**	**PXT3003 ID** **(** ***n*** **= 21)**	**PXT3003 HD** **(** ***n*** **= 19)**	**HD** ***versus*** **Placebo**		**HD** ***versus*** **PLI**	
**Non-deteriorated**	10 (52%)	10 (48%)	9 (43%)	15 (79%)	Relative Risk	*P*-value	Relative Risk	*P*-value
					1.5 (1.03;1.77)	0.047*	1.66 (1.24;1.93)	0.010*

Data are count (%) of non-deteriorated patients per group and Relative Risks (90% CI) for the HD group *versus* Placebo and *versus* PLI assessed with a logistic regression model. *P*-values are one-tailed. *P < 0.05.

## Discussion

Designing a clinical trial for CMT1A is a true medical challenge. This debilitating and heterogeneous rare disease has no approved reference treatment and its natural history has only recently begun to be systematically explored. The number of patients with confirmed diagnosis remains small, the disease progresses slowly and the relevance of efficacy outcomes remains an active topic of discussion, making it difficult to organise at this stage a study specifically powered for efficacy [45]. In this light, we consider the effects of PXT3003 described here as preliminary indications of drug activity, rather than definitive conclusions on drug efficacy.

Despite all these limitations, this study provides a set of relevant observations on the clinical measures, and gives estimates of the magnitude of improvement after one year of treatment under the doses tested.

**Figure 5:**
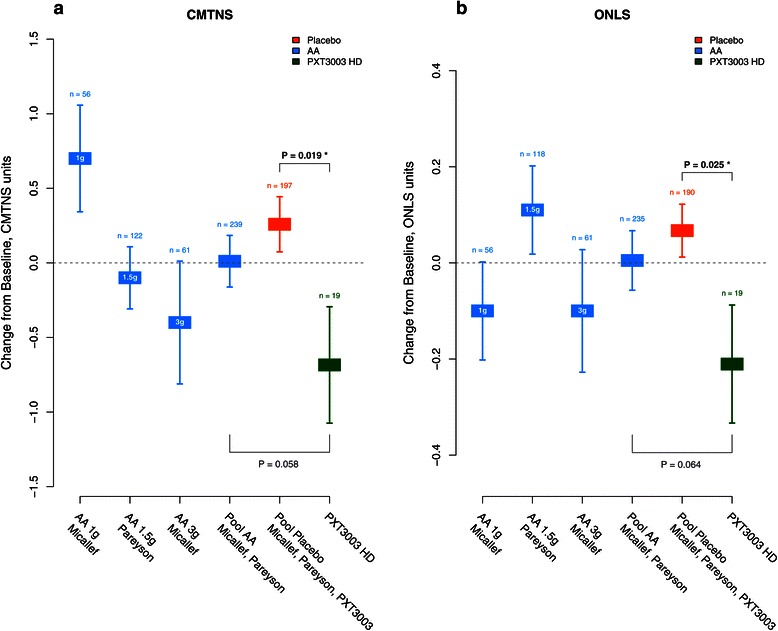
**Pooled analysis.** Mean change from baseline after 12 months (s.e.m.) of **(a)** CMTNS and **(b)** ONLS for single studies and pooled Placebo and ascorbic acid (AA) groups obtained from our study in addition to the French (Micallef *et al.* [16]) and Italian/UK (Pareyson *et al.* [17]) Phase 2 trials. For ONLS in Pareyson, values at 24 months were used, as values at 12 months were not available. Estimates of change at 12 months in previous trials were extracted from publications. *P < 0.05, *t*-test.

This study confirms the safety and tolerability of PXT3003. The highest dose shows consistent evidence of modest improvement after 12 months, not only with the data considered here but also in a comparison with a pool including 445 patients from published Ascorbic Acid studies suggesting the superiority of PXT3003 *versus* ascorbic acid (Figure 5). This preliminary result deserves further reporting in a proper and complete meta-analysis based on state-of-the-art methods.

The clinical composite scores CMTNS and ONLS, considered by us *a priori* as the two main endpoints, appeared to be the most responsive clinical outcomes despite their quasi stability after one year under Placebo. The improvement of the four functional measures (6MWT, Ankle dorsiflexion, Hand Grip and 9HPT) reached significance in the HD group compared to PLI (*P* = 0.051) in a multivariate analysis using O’Brien OLS test.

The evolution of Placebo subjects in our study and in previous CMT1A trials [13-18] underlines the fact that CMT1A patients deteriorate rather slowly. Shy *et al.* (2008) reported a natural deterioration of 0.686 points in CMTNS per year [46]. In this study we observed no such deterioration in patients under Placebo. The fact that patients under Placebo do not experience the natural progression of the disease was corroborated by previous clinical trials, and notably discussed by Pareyson *et al.* [17] and Lewis *et al.* [18]. The main conclusion is that natural history data of changes in the CMTNS cannot be used instead of a Placebo group in clinical trials. It has been suggested that an effective treatment for this disease should bring an improvement rather than the mere ability to slow the disease progression [18].

Actually, the most important improvement after one year in CMTNS and ONLS is observed in the HD group, while the changes observed under Placebo are negligible for CMTNS and with slight deterioration for ONLS (Figure 4 and Table 3). Nevertheless, the magnitude of improvement under treatment is rather small in this one-year trial and this does not permit us to draw definitive conclusions on the potential of PXT3003.

This promising finding could be interpreted through some mechanistic hypothetical considerations. From preclinical experiments it seems that the drug combination PXT3003, which hits potentially multiple targets, induces restoration and/or maintenance of Schwann cells and myelin in *Pmp22* rat model, and is also able to facilitate axon regeneration in a mouse nerve crush model (companion manuscript).

This pleiotropic effect could therefore ameliorate CMT1A patients through 3 different hypothetical mechanisms: *i)* axonal preservation which would delay or stabilise disease progression; *ii)* functional improvement of non-degenerated but dysfunctional suffering axons; *iii)* axonal regeneration. If axon preservation was the only mechanism, it would stabilise or delay disease progression, but because of the slow disease progression, assessing this effect would require several years of clinical observation. Alternatively, mechanisms *ii* and *iii* could explain an actual clinical amelioration of patients, instead of mere stabilisation, in a relatively short period of time.

The fact that electrophysiological measures of deterioration that are considered to reflect the state of myelin (see Methods) correlate with the clinical severity of the disease is one of the arguments favouring the possibility that a clinical amelioration could be linked to the functional improvement of dysmyelinated but preserved axons. In one of the first electrophysiological studies of 69 CMT1 patients [47], it was shown that peroneal nerve conduction velocity was significantly associated with neurologic disability score (NDS). But no such correlation was found in a cohort of 42 CMT1A patients, and it was concluded that only nerve action potentials (CMAP and SNAP), which reflect the degree of axonal loss, correlate with clinical severity. Still another group [48] reported a significant correlation between the clinical severity of the disease and the median nerve conduction velocity in a cohort of 51 CMT1A patients. In subsequent follow up of these patients, it was found that higher disability after 5 years is significantly associated with lower values of median nerve conduction velocity at baseline [49]. At least some of these discrepancies might be due to the differences and biases in patient recruitment. In our sample of 80 mild to moderate CMT1A patients (CMTNS ≤ 20), we also found that, at baseline, nerve conduction velocities were correlated with disease severity: correlations of MCV with CMTES and ONLS were respectively of −0.32 (*P* = 0.0067) and −0.37 (*P* = 0.0014).

Such correlation between MCV and disease severity, observed in three independent studies, should be confirmed in other studies. It may indicate that an improvement of myelin could, by itself, ameliorate CMT1A patient’s clinical state even if the degree of axonal loss remains unchanged. Actually, such therapeutic potential of PXT3003 on myelin is suggested by histological and electrophysiological outcomes after treatment not only of the *Pmp22* transgenic rat (companion manuscript) but also by the results on electrophysiological data observed in this trial.

The degree of such early therapeutic response, possibly related to a myelin improvement, should correlate with the proportion of dysfunctional but preserved axons. As this proportion is likely to be higher in patients with lower disease severity, this hypothesis fits with our observation that patients who have milder myelin impairment, as suggested by MCV and DML measures at baseline, seem to better respond to one-year treatment.

Moreover, PXT3003 was able to stimulate some axonal regeneration in acute nerve crush model as assessed by the amplitude of CMAP but the effect on this parameter in CMT1A transgenic rats was limited when the treatment was started after the onset of the disease (companion manuscript). It must be emphasized that in the present clinical trial, CMAP and SNAP amplitudes were measured at the distal part of the arm, innervated by long axonal fibres. However, a putative therapeutic axonal regeneration would be more likely to occur on shorter, i.e. more proximal, less affected fibres for which electrophysiological measures could not be performed in the present study. Still, such evidence based on electrophysiological parameters data must be taken cautiously because of the wide variability attached to these measures.

Despite its modest magnitude, the early amelioration observed by us could nevertheless be highly meaningful since it could announce a change of disease course by axon protection through a therapeutic action on myelin.

Obviously, our data suggest that even higher doses could be tested and might potentially generate greater amelioration. Longer treatment duration might also permit us to observe a larger improvement compared to Placebo through the various putative mechanisms discussed above.

## Conclusions

Taken together, these results suggest that PXT3003 combination deserves further clinical investigation. This might require more effort in assessing the specificity and the sensitivity of several outcomes. When designing a further trial in adults, it can be anticipated that efficacy would increase when including milder patients treated with higher dose and over a longer period. These results also suggest that PXT3003 could potentially be even more beneficial to children with a CMT1A genotype diagnosis, less affected than adults since much of the progression in CMT1A occurs during the first two decades of life [50].

In this regard, it would be tempting to test whether this treatment would be able to improve nerve conduction velocity in clinically unaffected CMT1A children. This would confirm a therapeutic action on myelin and would permit us to envision a preventive treatment in children, taking advantage of the quite safe profile of PXT3003 suggested by the nature and the low dosing of its compounds.

### Companion manuscript

Chumakov I, Milet A, Cholet N, Primas G, Boucard A, Pereira Y, Graudens E, Mandel J, Laffaire J, Foucquier J, Glibert F, Bertrand V, Nave KA, Sereda MW, Vial E, Guedj M, Hajj R, Nabirotchkin S, Cohen D. **Polytherapy with a combination of three repurposed drugs (PXT3003) down-regulates Pmp22 over-expression and improves myelination, axonal and functional parameters in models of CMT1A neuropathy.***Orphanet J Rare Dis* 2014, **9:**201.

## Additional files

Additional file 1: Table S1. PXT3003 daily doses (mg per day) per group. Additional file 2: Table S2. Baclofen concentrations in plasma. Additional file 3: Table S3. Minimum Effective Dose search performed by closed Step-Down procedure. Additional file 4: Table S4. Response to PXT3003 on efficacy outcomes (Full Analysis Set, *n* = 80). Additional file 5: Table S5. Characteristics of non-deteriorated and deteriorated patients at baseline (Full Analysis Set, *n* = 80).
