# Erratum to: An exploratory randomised double-blind and placebo-controlled phase 2 study of a combination of baclofen, naltrexone and sorbitol (PXT3003) in patients with Charcot-Marie-Tooth disease type 1A

**DOI:** 10.1186/s13023-016-0463-6

**Published:** 2016-07-07

**Authors:** Shahram Attarian, Jean-Michel Vallat, Laurent Magy, Benoît Funalot, Pierre-Marie Gonnaud, Arnaud Lacour, Yann Péréon, Odile Dubourg, Jean Pouget, Joëlle Micallef, Jérôme Franques, Marie-Noëlle Lefebvre, Karima Ghorab, Mahmoud Al-Moussawi, Vincent Tiffreau, Marguerite Preudhomme, Armelle Magot, Laurène Leclair-Visonneau, Tanya Stojkovic, Laura Bossi, Philippe Lehert, Walter Gilbert, Viviane Bertrand, Jonas Mandel, Aude Milet, Rodolphe Hajj, Lamia Boudiaf, Catherine Scart-Grès, Serguei Nabirotchkin, Mickael Guedj, Ilya Chumakov, Daniel Cohen

**Affiliations:** Centre de référence des maladies neuromusculaires et de la SLA, Pôle des neurosciences Cliniques, AP-HM et Aix Marseille Université, Marseille, France; CIC-Centre de Pharmacologie Clinique et D’Evaluations Therapeutiques, AP-HM et Aix Marseille Université, Marseille, France; CHU de Limoges - Hôpital Dupuytren, 2 Avenue Martin Luther King, 87042 Limoges, France; CHU Lyon Sud, 165 Chemin du Grand Revoyet, 69495 Lyon, France; CHRU de Lille - Hôpital Roger Salengro, rue Emile Laine, 59037 Lille, France; CHU de Nantes - Hôtel Dieu, 1 place Alexis Ricordeau, 44093 Nantes, France; CHU de Paris - Groupe Hospitalier Pitié-Salpétrière, 47-83 boulevard de l’Hôpital, 75013 Paris, France; Admissions, 75017 Paris, France; Faculty of Medicine, The University of Melbourne, Grattan St, Melbourne, VIC 3010 Australia; Faculty of Economics, UCL Mons, Louvain, Belgium; Carl M. Loeb University Professor Emeritus, Harvard University, Cambridge, MA 02138 USA; Pharnext, 11, rue des Peupliers, 92130 Issy-Les-Moulineaux, Paris, France

Unfortunately, the original version of this article [1] contained an error due to a typographical error in the computer code used. This meant the percentage improvement over baseline of the endpoints CMTNS, ONLS, 9HPT and DML was slightly increased. Therefore some of the values in Tables 3, 4, Additional file 4: Table S4 and Fig. 4 are incorrect.

**Table 3:** Response to PXT3003 on efficacy outcomes in treatment groups, with comparisons of active doses versus Placebo (Full Analysis Set, *n* = 80)

	Mean % of improvement				PXT3003 LD versus Placebo		PXT3003 ID versus Placebo		PXT3003 HD versus Placebo		Dose-effect	
	Placebo	PXT3003 LD	PXT3003 ID	PXT3003 HD	Estimate	*P*-value	Estimate	*P*-value	Estimate	*P*-value	Correlation	*P*-value
	(*n* = 19)	(*n* = 21)	(*n* = 21)	(*n* = 19)								
CMTNS	−0.25 (17.3)	−3.8 (20.4)	−5.8 (17.7)	**5.2 (12.5)**	−2.6 (−11.9;7.6)	0.67	−3.1 (−11.0;5.4)	0.74	5.5 (−3.4;15.2)	0.16	0.54	0.30
ONLS	−11.8 (33.7)	−12.7 (31.7)	1.2 (16.7)	**6.8 (18.2)**	−3.9 (−14.2;7.6)	0.72	6.9 (−3.8;18.8)	0.15	14.4 (0.55;30.2)	0.043*	0.28	0.006*
6MWT (m)	9.0 (8.3)	6.2 (8.3)	6.4 (9.4)	**9.9 (6.9)**	−2.4 (−6.2;1.5)	0.85	−2.4 (−6.6;2.0)	0.82	0.7 (−3.2;4.7)	0.38	0.11	0.16
9HPT (s)	3.6 (10.9)	−2.5 (12)	4.4 (9.5)	**6.1 (10.6)**	−4.6 (−10.3;1.5)	0.89	−0.2 (−5.3;5.2)	0.52	0.3 (−5.7;6.6)	0.47	0.15	0.092
Ankle Dorsiflexion (Nm)	20.2 (88.4)	−3.6 (43.0)	**81.5 (369.6)**	20.4 (64.1)	−4.0 (−21.7;17.8)	0.63	11.4 (−15.4;46.8)	0.26	8.2 (−13.8;35.9)	0.28	0.11	0.16
Grip (kg)	9.9 (24.2)	1.3 (15.6)	4.7 (12.5)	**11.7 (18.1)**	−7.1 (−15.6;2.1)	0.90	−3.6 (−11.8;5.4)	0.75	1.6 (−7.7;11.9)	0.39	0.12	0.15
CMAP (milliV)	34.4 (62.0)	1.4 (38.7)	22.9 (62.6)	**64.2 (208.5)**	−25.1 (−44.8;1.5)	0.94	−9.2 (−27.3;13.5)	0.77	−5.1 (−27.1;23.6)	0.63	−0.001	0.50
MCV (m/s)	3.7 (8.5)	3.0 (11.5)	5.7 (12.3)	**9.0 (17.6)**	−1.0 (−6.5;4.9)	0.61	0.5 (−4.8;6.2)	0.44	2.8 (−3.4;9.4)	0.23	0.11	0.18
DML (ms)	−0.33 (8.7)	0.33 (16.1)	**8.3 (18.1)**	5 (15.2)	3.4 (−4.3;11.7)	0.24	13.8 (4.2;24.3)	0.009*	8.0 (0.59;16.0)	0.038*	0.21	0.035*
SNAP (microV)	12.4 (121.7)	11.5 (88.2)	**23.3 (128.4)**	5.2 (69.0)	−1.2 (−42.9;71.0)	0.52	8.7 (−31.2;71.6)	0.38	13.9 (−24.1;71.0)	0.29	0.09	0.30
SCV (m/s)	3.4 (11.0)	5.3 (11.2)	29.5 (63.4)	**30.5 (10.0)**	1.5 (−5.8;9.4)	0.36	17.5 (−5.5;46.2)	0.11†	26.6 (15.5;38.8)	0.00037*	0.42	0.01*

Data are mean % (s.d.) of improvement for each treatment group. Differences between treatment groups were assessed by Analysis of Covariance (ANCOVA) on log-transformed values by adjusting for baseline values. Estimates were provided as mean percentage change over baseline (90 % CI). Dose-effect was tested through Spearman’s rank correlation. *P*-values are one-tailed. *P < 0.05; Shading = best improvement within dosages. *CMTNS* Charcot-Marie-Tooth Neuropathy Score, *ONLS* Overall Neuropathy Limitations Scale, *6MWT* 6-Minute Walk Test, *9HPT* 9-Hole Peg Test, *CMAP* Amplitudes of Compound Muscle Action Potentials, *MCV* Motor Conduction Velocity, *DML* Distal Motor Latency, *SNAP* Amplitudes of Sensory Nerve Action Potentials, *SCV* Sensitive Conduction Velocity

**Table 4:** Response to PXT3003 on efficacy outcomes in HD and in PLI, with comparisons of HD versus PLI (Full Analysis Set, *n* = 80)

	Mean % of improvement		PXT3003 HD versus PLI	
	PLI	PXT3003 HD	Estimate	*P*-value
	(*n* = 61)	(*n* = 19)		
CMTNS	−3.4 (18.4)	**5.2 (12.5)**	8.0 (0.4;16.2)	0.042*
ONLS	−7.7 (28.5)	**6.8 (18.2)**	12.1 (2.0;23.2)	0.024*
6MWT (m)	7.1 (8.6)	**9.9 (6.9)**	2.6 (−0.73;6.1)	0.099
9HPT (s)	1.8 (11.1)	**6.1 (10.6)**	1.2 (−3.4;6.0)	0.33
Ankle Dorsiflexion (Nm)	**33.1 (223.2)**	20.4 (64.1)	5.5 (−12.8;27.7)	0.32
Grip (kg)	5.1 (17.9)	**11.7 (18.1)**	6.0 (−1.2;13.7)	0.088
CMAP (milliV)	19.6 (56.5)	**64.2 (208.5)**	6.6 (−15.8;35.1)	0.33
MCV (m/s)	4.2 (10.9)	**9.0 (17.6)**	2.5 (−2.4;7.7)	0.21
DML (ms)	3 (15.3)	**5 (15.2)**	2.2 (−5.1;10.0)	0.31
SNAP (microV)	**15.9 (110.2)**	5.2 (69.0)	12.0 (−23.9;64.9)	0.31
SCV (m/s)	12.7 (38.0)	**30.5 (10.0)**	20.1 (2.4;40.8)	0.030*

Data are mean % (s.d.) of improvement for HD and for PLI after 12 months. Differences between treatment groups were assessed by Analysis of Covariance (ANCOVA) on log-transformed values by adjusting for baseline values. Estimates were provided as mean percentage change over baseline (90 % CI). *P*-values are one-tailed. *P < 0.05; Shading = best improvement within groups. *CMTNS* Charcot-Marie-Tooth Neuropathy Score, *ONLS* Overall Neuropathy Limitations Scale, *6MWT* 6-Minute Walk Test, *9HPT* 9-Hole Peg Test, *CMAP* Amplitudes of Compound Muscle Action Potentials, *MCV* Motor Conduction Velocity, *DML* Distal Motor Latency, *SNAP* Amplitudes of Sensory Nerve Action Potentials, *SCV* Sensitive Conduction Velocity

**Fig. 4:**
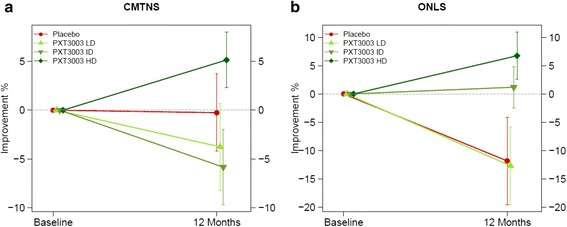
Response to PXT3003 on clinical scales (Full Analysis Set, *n* = 80). Mean % (s.e.m.) of improvement from baseline per group at 12 months for CMTNS (**a**) and ONLS (**b**). Sample sizes: Placebo (*n* = 19), LD (*n* = 21), ID (*n* = 21), HD (*n* = 19)

However this error has no bearing on statistical outcome of the study, the conclusions or text of the manuscript. Correct versions of Tables 3, 4 and Fig. 4 can be seen below and Additional file 4 accessed using the link below.

## Additional file

Additional file 4: Table S4. Response to PXT3003 on efficacy outcomes (Full Analysis Set, *n* = 80). Data are mean (s.d.) baseline and final values, and % (s.d.) of improvement for each treatment group and PLI. Differences between treatment groups were assessed by Analysis of Covariance (Ancova) on log-transformed values by adjusting for baseline values. Estimates were provided as mean percentage change over baseline (90 % CI). Dose-effect was tested through Spearman’s rank correlation. *P*-values are one-tailed. CMTNS = Charcot-Marie-Tooth Neuropathy Score; ONLS = Overall Neuropathy Limitations Scale; 6MWT = 6-Minute Walk Test; 9HPT = 9-Hole Peg Test; CMAP = Amplitudes of Compound Muscle Action Potentials; MCV = Motor Conduction Velocity; DML = Distal Motor Latency; SNAP = Amplitudes of Sensory Nerve Action Potentials; SCV = Sensitive Conduction Velocity; VAS = Visual Analog Scale; CGI = Clinical Global Impression. (DOC 168 kb)
