# Supplementary material for: An exploratory randomised double-blind and placebo-controlled phase 2 study of a combination of baclofen, naltrexone and sorbitol (PXT3003) in patients with Charcot-Marie-Tooth disease type 1A
Source: Orphanet J Rare Dis. 2014 Dec 18;9:199. doi: 10.1186/s13023-014-0199-0 (PMC4311411; doi:10.1186/s13023-014-0199-0)
Supplement: Additional file 1: Table S1. — PXT3003 daily doses (mg per day) per group. [file 13023_2014_199_MOESM1_ESM.pdf]

Additional Table 1 | PXT3003 daily doses (mg per day) per group.

PXT3003	Baclofen	Naltrexone	Sorbitol	Dilution
Low dose (LD)	0.6	0.07	21	1/10
Intermediate dose (ID)	1.2	0.14	42	1/5
High dose (HD)	6	0.7	210	1
**Authorized recommended dose**	60 *(spasticity)*	50 *(alcohol dependence)*	15 000 (aperient)	
