# Supplementary material for: An exploratory randomised double-blind and placebo-controlled phase 2 study of a combination of baclofen, naltrexone and sorbitol (PXT3003) in patients with Charcot-Marie-Tooth disease type 1A
Source: Orphanet J Rare Dis. 2014 Dec 18;9:199. doi: 10.1186/s13023-014-0199-0 (PMC4311411; doi:10.1186/s13023-014-0199-0)
Supplement: Additional file 2: Table S2. — Baclofen concentrations in plasma. [file 13023_2014_199_MOESM2_ESM.pdf]

Additional Table 2 | Baclofen concentrations in plasma, expressed in ng/mL (s.d.).

Time	PXT3003 LD		PXT3003 ID		PXT3003 HD	
	Trough	Peak	Trough	Peak	Trough	Peak
**1 month**	0.7 (0.5)	4.7 (1.3)	1.8 (2.2)	9.9 (3.1)	7.7 (4.6)	49.9 (13.5)
**6 months**	1.3 (1.8)	4.1 (1.8)	4.1 (10.7)	13.8 (12)	9.4 (9.6)	47.0 (21.1)
**12 months**	0.7 (0.4)	5.3 (2.2)	2.0 (2.5)	10.7 (5.2)	8.2 (6.0)	51.8 (19.8)
