# Supplementary material for: An exploratory randomised double-blind and placebo-controlled phase 2 study of a combination of baclofen, naltrexone and sorbitol (PXT3003) in patients with Charcot-Marie-Tooth disease type 1A
Source: Orphanet J Rare Dis. 2014 Dec 18;9:199. doi: 10.1186/s13023-014-0199-0 (PMC4311411; doi:10.1186/s13023-014-0199-0)
Supplement: Additional file 3: Table S3. — Minimum Effective Dose search performed by closed Step-Down procedure. [file 13023_2014_199_MOESM3_ESM.pdf]

Additional Table 3 | Minimum Effective Dose search performed by closed Step-Down procedure for comparing ordered doses with a control (S2DH algorithm). *$P < 0.05$.

Contrasts				
**Placebo**	**PXT3003 LD**	**PXT3003 ID**	**PXT3003 HD**	***P*-value O'Brien(CMTNS,ONLS)**
-1	1	0	0	0.969
-1	-1	2	0	0.513
-1	-1	-1	3	0.006*
