# Supplementary material for: An exploratory randomised double-blind and placebo-controlled phase 2 study of a combination of baclofen, naltrexone and sorbitol (PXT3003) in patients with Charcot-Marie-Tooth disease type 1A
Source: Orphanet J Rare Dis. 2014 Dec 18;9:199. doi: 10.1186/s13023-014-0199-0 (PMC4311411; doi:10.1186/s13023-014-0199-0)
Supplement: Additional file 4: Table S4. — Response to PXT3003 on efficacy outcomes (Full Analysis Set, n = 80). [file 13023_2014_199_MOESM4_ESM.pdf]

Additional Table 4 | Response to PXT3003 on efficacy outcomes (Full Analysis Set, *n* = 80). Data are mean (s.d.) baseline and final values, and % (s.d.) of improvement for each treatment group and PLI. Differences between treatment groups were assessed by Analysis of Covariance (Ancova) on log-transformed values by adjusting for baseline values. Estimates were provided as mean percentage change over baseline (90% CI). Dose-effect was tested through Spearman's rank correlation. *P*-values are one-tailed. CMTNS = Charcot-Marie-Tooth Neuropathy Score; ONLS = Overall Neuropathy Limitations Scale; 6MWT = 6-Minute Walk Test; 9HPT = 9-Hole Peg Test; CMAP = Amplitudes of Compound Muscle Action Potentials; MCV = Motor Conduction Velocity; DML = Distal Motor Latency; SNAP = Amplitudes of Sensory Nerve Action Potentials; SCV = Sensitive Conduction Velocity; VAS = Visual Analog Scale; CGI = Clinical Global Impression.

	**Placebo** (*n* = 19)			**PXT3003 LD** (*n* = 21)			**PXT3003 ID** (*n* = 21)			**PXT3003 HD** (*n* = 19)		
	**Baseline**	**Final**	**% Improvement**	**Baseline**	**Final**	**% Improvement**	**Baseline**	**Final**	**% Improvement**	**Baseline**	**Final**	**% Improvement**
**CMTNS**	14.3 (3.8)	14.2 (3.8)	2.6 (17.5)	14.2 (4.1)	14.5 (4.2)	0.5 (23.2)	13.0 (4.0)	13.5 (3.7)	-3.1 (16.0)	13.8 (3.4)	13.2 (3.9)	7.7 (18.4)
**CMTES**	9.5 (3.0)	9.4 (2.7)	3.8 (25.1)	9.5 (3.4)	9.9 (3.3)	-2.1 (25.0)	8.8 (2.9)	9.1 (3.1)	-2.0 (20.0)	8.8 (2.7)	8.5 (2.7)	6.8 (21.8)
**CMTNS: Sensory Symptoms**	1.8 (1.1)	1.6 (0.8)	26.3 (87.2)	2.1 (1.1)	2.1 (1.2)	19.4 (84.2)	1.5 (0.7)	1.6 (0.7)	-0.8 (29.1)	1.7 (0.8)	1.4 (0.6)	36.0 (79.0)
**CMTNS: Motor Symptoms: Legs**	2.4 (0.8)	2.3 (0.7)	5.3 (15.8)	2.3 (0.6)	2.3 (0.7)	4.0 (26.8)	2.2 (0.8)	2.1 (0.9)	17.5 (41.7)	2.1 (0.5)	2.1 (0.5)	0.0 (0.0)
**CMTNS: Motor Symptoms: Arms**	1.7 (0.8)	1.6 (0.6)	5.3 (22.9)	1.9 (0.6)	1.8 (0.5)	4.8 (26.9)	1.8 (0.5)	1.7 (0.6)	11.9 (38.4)	1.7 (0.6)	1.6 (0.6)	5.3 (22.9)
**CMTNS: Pin Sensitivity**	2.5 (1.0)	2.4 (0.8)	10.5 (55.3)	2.3 (1.0)	2.1 (0.9)	27.8 (78.2)	2.4 (1.1)	2.6 (0.9)	-3.6 (35.4)	2.2 (0.9)	2.1 (0.9)	21.9 (74.3)
**CMTNS: Vibration Sensitivity**	2.8 (0.9)	3.3 (1.3)	-4.7 (35.2)	2.8 (1.0)	3.3 (1.2)	-11.2 (28.7)	2.7 (1.2)	3.2 (1.1)	-11.1 (38.4)	2.7 (0.7)	3.0 (1.0)	-3.2 (27.0)
**CMTNS: Strength: Legs**	2.7 (0.7)	2.6 (0.7)	5.3 (19.3)	2.6 (0.8)	2.6 (0.7)	0.0 (21.9)	2.6 (0.7)	2.6 (0.7)	0.8 (13.4)	2.8 (1.0)	2.6 (0.9)	9.2 (19.0)
**CMTNS: Strength: Arms**	2.5 (0.6)	2.6 (0.6)	0.0 (21.5)	2.6 (0.9)	2.7 (0.7)	-0.4 (30.1)	2.4 (0.9)	2.3 (0.8)	4.4 (18.9)	2.6 (1.1)	2.7 (0.8)	-3.9 (20.5)
**CMTNS: CMAP**	2.7 (0.9)	2.6 (1.0)	7.9 (27.4)	2.7 (0.9)	2.6 (0.8)	6.3 (37.7)	2.4 (1.0)	2.2 (0.9)	11.9 (30.8)	2.8 (1.0)	2.6 (0.9)	8.8 (24.4)
**CMTNS: SNAP**	4.1 (1.3)	4.2 (1.2)	-2.1 (20.8)	4.0 (1.2)	4.0 (1.4)	13.8 (53.6)	3.9 (1.4)	4.1 (1.3)	-1.0 (42.1)	4.2 (1.1)	4.1 (1.0)	4.1 (28.5)
**ONLS**	3.1 (1.1)	3.3 (0.9)	-5.3 (19.3)	3.3 (1.0)	3.6 (0.9)	-6.5 (18.5)	3.5 (0.9)	3.4 (0.9)	4.8 (24.2)	3.6 (0.8)	3.4 (1.0)	12.3 (28.4)
**ONLS: Arm**	2.4 (0.8)	2.4 (0.8)	3.5 (18.1)	2.7 (0.7)	2.9 (0.7)	-4.4 (11.1)	2.7 (0.7)	2.7 (0.7)	7.1 (45.5)	2.7 (0.7)	2.5 (0.8)	20.2 (37.9)
**ONLS: Leg**	2.7 (0.5)	2.9 (0.3)	-7.0 (14.0)	2.6 (0.5)	2.8 (0.4)	-4.0 (18.2)	2.8 (0.4)	2.7 (0.5)	2.4 (10.9)	2.8 (0.4)	2.9 (0.3)	-1.8 (7.6)
**6MWT (m)**	468.2 (99.9)	509.2 (107.7)	9.0 (8.3)	473.1 (70.9)	500.9 (75.7)	6.2 (8.3)	450.7 (71.1)	481.4 (95.2)	6.4 (9.4)	429.3 (83.7)	472.0 (98.0)	9.9 (6.9)
**9HPT (s)**	17.2 (2.5)	16.6 (3.0)	4.9 (11.4)	16.1 (3.9)	16.3 (3.5)	-1.2 (11.7)	18.4 (4.7)	17.4 (3.8)	5.6 (9.9)	20.8 (7.8)	19.1 (5.9)	7.8 (12.1)
**Ankle Dorsiflexion (Nm)**	7.8 (6.6)	7.4 (5.6)	20.2 (88.4)	9.1 (5.0)	7.7 (3.6)	-3.6 (43.0)	8.3 (5.6)	8.3 (5.1)	81.5 (369.6)	8.2 (6.1)	7.9 (5.2)	20.4 (64.1)
**Grip (kg)**	22.6 (10.7)	24.5 (12.0)	9.9 (24.2)	21.6 (6.1)	22.1 (7.8)	1.3 (15.6)	23.1 (9.2)	24.0 (9.3)	4.7 (12.5)	20.6 (10.4)	22.4 (10.3)	11.7 (18.1)
**CMAP (milliV)**	3.7 (2.0)	4.4 (2.0)	34.4 (62.0)	4.0 (1.8)	3.5 (1.7)	1.4 (38.7)	3.7 (2.1)	4.0 (2.2)	22.9 (62.6)	3.4 (2.3)	3.9 (2.1)	64.2 (208.5)
**MCV (m/s)**	21.5 (3.6)	22.4 (4.7)	3.7 (8.5)	22.7 (4.7)	22.5 (5.6)	3.0 (11.5)	20.8 (4.8)	21.6 (3.8)	5.7 (12.3)	20.5 (5.3)	21.6 (4.8)	9.0 (17.6)
**DML (ms)**	8.6 (2.2)	8.6 (2.3)	0.4 (8.8)	7.9 (2.1)	7.9 (2.0)	3.6 (21.7)	8.2 (1.8)	7.3 (1.3)	15.3 (35.8)	8.2 (1.9)	7.6 (1.4)	8.4 (21.7)
**SNAP (microV)**	2.6 (3.2)	2.6 (2.9)	12.4 (121.7)	2.3 (3.0)	3.0 (3.7)	11.5 (88.2)	2.6 (3.8)	2.8 (3.6)	23.3 (128.4)	2.2 (2.7)	2.5 (2.7)	5.2 (69.0)
**SCV (m/s)**	31.1 (14.8)	31.3 (12.1)	3.4 (11.0)	29.4 (8.2)	30.9 (7.5)	5.3 (11.2)	31.3 (9.4)	33.9 (9.1)	29.5 (63.4)	29.9 (7.7)	35.8 (10.4)	30.5 (10.0)
**VAS: Pain**	83.9 (23.1)	80.8 (21.2)	0.8 (32.0)	85.6 (16.8)	70.9 (23.7)	-16.7 (23.4)	82.0 (17.5)	84.3 (16.7)	9.1 (41.3)	80.9 (22.2)	71.1 (24.0)	-6.7 (44.3)
**VAS: Fatigue**	61.6 (27.4)	65.8 (26.3)	29.8 (80.3)	70.0 (20.8)	57.1 (26.1)	-17.4 (33.7)	70.2 (18.6)	61.2 (22.3)	-6.8 (40.5)	62.6 (29.6)	57.3 (26.4)	-1.0 (39.7)
**VAS: Global**	65.5 (25.3)	74.7 (18.6)	51.3 (152.4)	72.5 (18.9)	71.4 (18.5)	1.8 (27.8)	64.4 (27.5)	71.4 (21.2)	62.3 (161.6)	78.4 (16.5)	70.8 (19.8)	-8.1 (26.3)
**CGI: Global Improvement**	4.0 (0.0)	4.1 (0.2)	-1.1 (4.6)	4.0 (0.0)	4.0 (0.3)	0.6 (8.7)	4.0 (0.0)	3.7 (0.5)	9.5 (15.4)	4.0 (0.0)	4.1 (0.3)	-2.1 (6.3)
**CGI: Illness Severity**	3.5 (1.1)	3.4 (1.1)	5.9 (27.6)	3.5 (0.8)	3.5 (0.9)	12.7 (68.0)	3.5 (0.7)	3.7 (1.1)	-1.7 (23.9)	3.7 (0.9)	3.8 (0.9)	-2.6 (16.4)
**CGI: Therapeutic Effect**	4.0 (0.0)	3.9 (0.2)	1.8 (7.6)	4.0 (0.0)	4.0 (0.2)	1.6 (7.3)	4.0 (0.0)	3.7 (0.6)	12.7 (24.7)	4.0 (0.0)	4.0 (0.0)	0.0 (0.0)

	PLI (*n* = 61)			**PXT3003 LD *versus* Placebo**		**PXT3003 ID *versus* Placebo**		**PXT3003 HD *versus* Placebo**		**PXT3003 HD *versus* PLI**		**Dose-effect**	
	**Baseline**	**Final**	**% Improvement**	**Estimate**	***P*-value**	**Estimate**	***P*-value**	**Estimate**	***P*-value**	**Estimate**	***P*-value**	**Correlation**	***P*-value**
**CMTNS**	13.9 (4.0)	14.0 (3.9)	-0.1 (19.0)	-2.6 (-11.9;7.6)	0.67	-3.1 (-11.0;5.4)	0.74	5.5 (-3.4;15.2)	0.16	8.0 (0.4;16.2)	0.042	0.06	0.3
**CMTES**	9.3 (3.1)	9.5 (3.0)	-0.2 (23.2)	-4.9 (-15.4;6.9)	0.76	-1.9 (-12.1;9.4)	0.62	6.3 (-5.3;19.2)	0.19	8.7 (-0.39;18.6)	0.058	0.034	0.38
**CMTNS: Sensory Symptoms**	1.8 (1.0)	1.8 (1.0)	14.6 (71.1)	-13.0 (-31.8;10.9)	0.83	-6.6 (-23.7;14.2)	0.72	14.0 (-8.1;41.3)	0.16	21.0 (1.4;44.3)	0.038	0.081	0.24
**CMTNS: Motor Symptoms: Legs**	2.3 (0.7)	2.2 (0.8)	9.0 (30.5)	-2.6 (-11.6;7.3)	0.68	7.4 (-5.5;22.1)	0.18	-3.3 (-8.0;1.7)	0.87	-5.3 (-13.4;3.6)	0.84	-0.038	0.63
**CMTNS: Motor Symptoms: Arms**	1.8 (0.6)	1.7 (0.6)	7.4 (30.1)	-5.1 (-15.1;6.2)	0.78	-0.1 (-12.6;14.2)	0.51	-0.007 (-9.5;10.5)	0.5	1.1 (-8.2;11.4)	0.42	-0.02	0.57
**CMTNS: Pin Sensitivity**	2.4 (1.0)	2.4 (0.9)	11.6 (59.6)	18.1 (-3.9;45.2)	0.091	-6.4 (-20.6;10.4)	0.75	15.3 (-6.3;42.0)	0.13	9.4 (-7.5;29.4)	0.19	-0.056	0.69
**CMTNS: Vibration Sensitivity**	2.8 (1.0)	3.3 (1.2)	-9.2 (33.9)	-5.1 (-20.6;13.5)	0.69	-3.8 (-19.4;14.8)	0.64	4.8 (-12.2;25.1)	0.33	8.6 (-5.3;24.4)	0.16	0.017	0.44
**CMTNS: Strength: Legs**	2.6 (0.8)	2.6 (0.7)	1.9 (18.3)	-3.7 (-12.8;6.2)	0.74	-2.8 (-10.1;5.2)	0.73	3.6 (-5.0;13.0)	0.25	6.1 (-1.2;13.9)	0.084	0.085	0.23
**CMTNS: Strength: Arms**	2.5 (0.8)	2.5 (0.7)	1.4 (23.8)	-2.4 (-14.2;11.0)	0.63	7.1 (-3.0;18.2)	0.12	-3.6 (-12.5;6.2)	0.73	-5.5 (-14.0;3.8)	0.84	0.012	0.46
**CMTNS: CMAP**	2.6 (0.9)	2.5 (0.9)	8.7 (32.0)	-3.5 (-16.4;11.4)	0.66	4.8 (-7.9;19.1)	0.27	0.2 (-11.1;13.0)	0.49	-0.8 (-11.0;10.7)	0.55	0.089	0.22
**CMTNS: SNAP**	4.0 (1.3)	4.1 (1.3)	3.7 (41.6)	9.9 (-7.1;30.0)	0.17	-1.4 (-16.7;16.7)	0.56	3.2 (-8.3;16.1)	0.33	0.7 (-12.5;15.9)	0.47	0.024	0.42
**ONLS**	3.3 (1.0)	3.4 (0.9)	-2.2 (21.2)	-3.9 (-14.2;7.6)	0.72	6.9 (-3.8;18.8)	0.15	14.4 (0.55;30.2)	0.043	12.1 (2.0;23.2)	0.024	0.28	0.0059
**ONLS: Arm**	2.6 (0.8)	2.6 (0.8)	2.0 (29.2)	-8.1 (-15.2;-0.41)	0.96	-2.8 (-14.2;10.2)	0.65	13.7 (0.22;29.0)	0.047	15.7 (4.9;27.6)	0.0076	0.22	0.027
**ONLS: Leg**	2.7 (0.5)	2.8 (0.4)	-2.7 (15.0)	4.5 (-3.0;12.5)	0.16	9.7 (3.1;16.7)	0.0078	3.0 (-2.7;9.0)	0.19	-0.9 (-6.2;4.7)	0.61	0.21	0.03
**6MWT (m)**	463.8 (80.0)	496.6 (92.1)	7.1 (8.6)	-2.4 (-6.2;1.5)	0.85	-2.4 (-6.6;2.0)	0.82	0.7 (-3.2;4.7)	0.38	2.6 (-0.73;6.1)	0.099	0.11	0.16
**9HPT (s)**	17.2 (3.9)	16.8 (3.4)	3.1 (11.3)	-4.6 (-10.3;1.5)	0.89	-0.2 (-5.3;5.2)	0.52	0.3 (-5.7;6.6)	0.47	1.2 (-3.4;6.0)	0.33	0.15	0.092
**Ankle Dorsiflexion (Nm)**	8.4 (5.7)	7.8 (4.7)	33.1 (223.2)	-4.0 (-21.7;17.8)	0.63	11.4 (-15.4;46.8)	0.26	8.2 (-13.8;35.9)	0.28	5.5 (-12.8;27.7)	0.32	0.11	0.16
**Grip (kg)**	22.4 (8.7)	23.5 (9.7)	5.1 (17.9)	-7.1 (-15.6;2.1)	0.9	-3.6 (-11.8;5.4)	0.75	1.6 (-7.7;11.9)	0.39	6.0 (-1.2;13.7)	0.088	0.12	0.15
**CMAP (milliV)**	3.8 (2.0)	4.0 (2.0)	19.6 (56.5)	-25.1 (-44.8;1.5)	0.94	-9.2 (-27.3;13.5)	0.77	-5.1 (-27.1;23.6)	0.63	6.6 (-15.8;35.1)	0.33	-0.0011	0.5
**MCV (m/s)**	21.6 (4.4)	22.2 (4.7)	4.2 (10.9)	-1.0 (-6.5;4.9)	0.61	0.5 (-4.8;6.2)	0.44	2.8 (-3.4;9.4)	0.23	2.5 (-2.4;7.7)	0.21	0.11	0.18
**DML (ms)**	8.2 (2.0)	7.9 (2.0)	6.7 (25.6)	3.4 (-4.3;11.7)	0.24	13.8 (4.2;24.3)	0.0092	8.0 (0.59;16.0)	0.038	2.2 (-5.1;10.0)	0.31	0.21	0.035
**SNAP (microV)**	2.5 (3.3)	2.8 (3.4)	15.9 (110.2)	-1.2 (-42.9;71.0)	0.52	8.7 (-31.2;71.6)	0.38	13.9 (-24.1;71.0)	0.29	12.0 (-23.9;64.9)	0.31	0.089	0.3
**SCV (m/s)**	30.6 (10.7)	32.0 (9.2)	12.7 (38.0)	1.5 (-5.8;9.4)	0.36	17.5 (-5.5;46.2)	0.11	26.6 (15.5;38.8)	0.00037	20.1 (2.4;40.8)	0.03	0.42	0.0098
**VAS: Pain**	83.9 (18.9)	78.6 (21.2)	-2.4 (34.4)	-17.4 (-30.7;-1.6)	0.96	6.7 (-7.2;22.6)	0.22	-13.6 (-29.0;5.3)	0.89	-10.3 (-23.2;4.8)	0.88	-0.057	0.69
**VAS: Fatigue**	67.5 (22.4)	61.2 (24.7)	1.0 (57.1)	-25.6 (-43.1;-2.7)	0.96	-10.5 (-28.8;12.6)	0.79	-17.0 (-34.4;5.0)	0.91	-2.5 (-20.3;19.3)	0.58	-0.094	0.8
**VAS: Global**	67.5 (24.0)	72.4 (19.2)	38.0 (129.0)	-8.1 (-21.0;6.9)	0.82	-11.4 (-33.5;18.0)	0.76	-10.5 (-24.5;6.1)	0.86	-2.6 (-19.4;17.7)	0.59	-0.21	0.97
**CGI: Global Improvement**	4.0 (0.0)	3.9 (0.4)	3.2 (11.5)	1.5 (-2.2;5.3)	0.25	9.8 (4.0;16.0)	0.0032	-1.2 (-4.4;2.2)	0.72	-4.8 (-8.7;-0.69)	0.97	0.051	0.33
**CGI: Illness Severity**	3.5 (0.8)	3.5 (1.0)	5.6 (44.7)	-1.4 (-15.6;15.2)	0.56	-8.1 (-18.9;4.1)	0.87	-8.3 (-17.5;1.9)	0.91	-6.1 (-16.0;5.0)	0.82	-0.15	0.91
**CGI: Therapeutic Effect**	4.0 (0.0)	3.9 (0.4)	5.5 (16.3)	-0.1 (-3.5;3.3)	0.53	9.0 (1.1;17.5)	0.03	-1.5 (-4.0;1.0)	0.84	-4.3 (-8.8;0.36)	0.94	0.042	0.35
