# Supplementary material for: An exploratory randomised double-blind and placebo-controlled phase 2 study of a combination of baclofen, naltrexone and sorbitol (PXT3003) in patients with Charcot-Marie-Tooth disease type 1A
Source: Orphanet J Rare Dis. 2014 Dec 18;9:199. doi: 10.1186/s13023-014-0199-0 (PMC4311411; doi:10.1186/s13023-014-0199-0)
Supplement: Additional file 5: Table S5. — Characteristics of non-deteriorated and deteriorated patients at baseline (Full Analysis Set, n = 80). [file 13023_2014_199_MOESM5_ESM.pdf]

Additional Table 5 | Characteristics of non-deteriorated and deteriorated patients at baseline (Full Analysis Set, *n* = 80). Data are mean (s.d.) values at baseline. *P*-values of between-group differences at baseline were assessed by Anova. **P* < 0.05; Shading = best mean value at baseline within non-deteriorated and deteriorated patients. CMTNS = Charcot-Marie-Tooth Neuropathy Score; ONLS = Overall Neuropathy Limitations Scale; 6MWT = 6-Minute Walk Test; 9HPT = 9-Hole Peg Test; CMAP = Amplitudes of Compound Muscle Action Potentials; MCV = Motor Conduction Velocity; DML = Distal Motor Latency; SNAP = Amplitudes of Sensory Nerve Action Potentials; SCV = Sensitive Conduction Velocity.

	**a - Baseline in PXT3003 HD**			**b - Baseline in Placebo**		
	**Non-deteriorated** (*n* = 15)	**Deteriorated** (*n* = 4)	***P*-value**	**Non-deteriorated** (*n* = 10)	**Deteriorated** (*n* = 9)	***P*-value**
**CMTNS**	13.3 (3.2)	16.0 (3.6)	0.16	15.0 (3.1)	13.6 (4.6)	0.42
**ONLS**	3.5 (0.8)	3.8 (0.5)	0.63	3.3 (0.8)	2.9 (1.4)	0.43
**6MWT (m)**	448.3 (67.8)	358.0 (109.5)	0.052	446.3 (97.3)	490.2 (103.3)	0.37
**9HPT (s)**	20.5 (7.9)	21.9 (8.3)	0.76	16.8 (2.3)	17.6 (2.7)	0.46
**Ankle Dorsiflexion (Nm)**	8.0 (5.5)	8.9 (8.8)	0.79	7.7 (8.0)	7.9 (5.0)	0.94
**Grip (kg)**	22.5 (10.4)	13.5 (7.8)	0.13	23.1 (11.2)	21.9 (10.7)	0.81
**CMAP (milliV)**	3.8 (2.0)	1.8 (3.1)	0.14	4.0 (1.9)	3.3 (2.2)	0.47
**MCV (m/s)**	22.5 (3.5)	14.7 (5.7)	0.0051*	21.1 (4.1)	22.1 (3.0)	0.58
**DML (ms)**	7.6 (1.4)	10.2 (2.4)	0.010*	8.4 (1.3)	8.9 (3.0)	0.64
**SNAP (microV)**	2.4 (2.8)	1.4 (2.8)	0.54	2.7 (3.4)	2.5 (3.1)	0.91
**SCV (m/s)**	30.3 (8.6)	28.2 (NA)	0.83	24.4 (5.1)	35.1 (17.8)	0.36
