# Supplementary material for: Erratum to: An exploratory randomised double-blind and placebo-controlled phase 2 study of a combination of baclofen, naltrexone and sorbitol (PXT3003) in patients with Charcot-Marie-Tooth disease type 1A
Source: Orphanet J Rare Dis. 2016 Jul 7;11:92. doi: 10.1186/s13023-016-0463-6 (PMC4937550; doi:10.1186/s13023-016-0463-6)
Supplement: Additional file 4: Table S4. — Response to PXT3003 on efficacy outcomes (Full Analysis Set, n = 80). Data are mean (s.d.) baseline and final values, and % (s.d.) of improvement for each treatment group and PLI. Differences between treatment groups were assessed by Analysis of Covariance (Ancova) on log-transformed values by adjusting for baseline values. Estimates were provided as mean percentage change over baseline (90 % CI). Dose-effect was tested through Spearman’s rank correlation. P-values are one-tailed. CMTNS = Charcot-Marie-Tooth Neuropathy Score; ONLS = Overall Neuropathy Limitations Scale; 6MWT = 6-Minute Walk Test; 9HPT = 9-Hole Peg Test; CMAP = Amplitudes of Compound Muscle Action Potentials; MCV = Motor Conduction Velocity; DML = Distal Motor Latency; SNAP = Amplitudes of Sensory Nerve Action Potentials; SCV = Sensitive Conduction Velocity; VAS = Visual Analog Scale; CGI = Clinical Global Impression. (DOC 168 kb) [file 13023_2016_463_MOESM1_ESM.doc]

**Additional Table 4 | Response to PXT3003 on efficacy outcomes (Full Analysis Set, *n* = 80).** Data are mean (s.d.) baseline and final values, and % (s.d.) of improvement for each treatment group and PLI. Differences between treatment groups were assessed by Analysis of Covariance (Ancova) on log-transformed values by adjusting for baseline values. Estimates were provided as mean percentage change over baseline (90% CI). Dose-effect was tested through Spearman’s rank correlation. *P*-values are one-tailed. CMTNS = Charcot-Marie-Tooth Neuropathy Score; ONLS = Overall Neuropathy Limitations Scale; 6MWT = 6-Minute Walk Test; 9HPT = 9-Hole Peg Test; CMAP = Amplitudes of Compound Muscle Action Potentials; MCV = Motor Conduction Velocity; DML = Distal Motor Latency; SNAP = Amplitudes of Sensory Nerve Action Potentials; SCV = Sensitive Conduction Velocity; VAS = Visual Analog Scale; CGI = Clinical Global Impression.

|  | **Placebo**  (*n* = 19) | | | **PXT3003 LD**  (*n* = 21) | | | | | **PXT3003 ID**  (*n* = 21) | | | | | **PXT3003 HD**  (*n* = 19) | | | | |
| --- | --- | --- | --- | --- | --- | --- | --- | --- | --- | --- | --- | --- | --- | --- | --- | --- | --- | --- |
|  | **Baseline** | **Final** | **%**  **Improvement** | | | **Baseline** | **Final** | **%**  **Improvement** | | | **Baseline** | **Final** | **%**  **Improvement** | | **Baseline** | **Final** | **%**  **Improvement** | |
| **CMTNS** | 14.3 (3.8) | 14.2 (3.8) | -0.25 (17.3) | | 14.2 (4.1) | | 14.5 (4.2) | -3.8 (20.4) | | 13.0 (4.0) | | 13.5 (3.7) | -5.8 (17.7) | | 13.8 (3.4) | 13.2 (3.9) | | 5.2 (12.5) |
| **CMTES** | 9.5 (3.0) | 9.4 (2.7) | -2.6 (28.3) | | 9.5 (3.4) | | 9.9 (3.3) | -7.6 (24.1) | | 8.8 (2.9) | | 9.1 (3.1) | -5.1 (16.5) | | 8.8 (2.7) | 8.5 (2.7) | | 3.4 (15.7) |
| **CMTNS: Sensory Symptoms** | 1.8 (1.1) | 1.6 (0.8) | -4 (49.1) | | 2.1 (1.1) | | 2.1 (1.2) | -9.9 (52.7) | | 1.5 (0.7) | | 1.6 (0.7) | -11.9 (49.8) | | 1.7 (0.8) | 1.4 (0.6) | | 4.4 (56.1) |
| **CMTNS: Motor Symptoms: Legs** | 2.4 (0.8) | 2.3 (0.7) | 3.5 (10.5) | | 2.3 (0.6) | | 2.3 (0.7) | -0.79 (20.7) | | 2.2 (0.8) | | 2.1 (0.9) | 7.1 (23.9) | | 2.1 (0.5) | 2.1 (0.5) | | 0 (0) |
| **CMTNS: Motor Symptoms: Arms** | 1.7 (0.8) | 1.6 (0.6) | 0 (27.2) | | 1.9 (0.6) | | 1.8 (0.5) | -0.79 (26.1) | | 1.8 (0.5) | | 1.7 (0.6) | 2.4 (29.5) | | 1.7 (0.6) | 1.6 (0.6) | | 2.6 (11.5) |
| **CMTNS: Pin Sensitivity** | 2.5 (1.0) | 2.4 (0.8) | -8.3 (46.9) | | 2.3 (1.0) | | 2.1 (0.9) | 0 (48.1) | | 2.4 (1.1) | | 2.6 (0.9) | -17.9 (45.4) | | 2.2 (0.9) | 2.1 (0.9) | | -6.1 (50.4) |
| **CMTNS: Vibration Sensitivity** | 2.8 (0.9) | 3.3 (1.3) | -19.3 (48) | | 2.8 (1.0) | | 3.3 (1.2) | -28.2 (53.9) | | 2.7 (1.2) | | 3.2 (1.1) | -31.4 (51.4) | | 2.7 (0.7) | 3.0 (1.0) | | -12.7 (40.3) |
| **CMTNS: Strength: Legs** | 2.7 (0.7) | 2.6 (0.7) | 2.2 (17.1) | | 2.6 (0.8) | | 2.6 (0.7) | -4.6 (23.2) | | 2.6 (0.7) | | 2.6 (0.7) | -0.79 (13.4) | | 2.8 (1.0) | 2.6 (0.9) | | 6.3 (12.9) |
| **CMTNS: Strength: Arms** | 2.5 (0.6) | 2.6 (0.6) | -4.4 (22.8) | | 2.6 (0.9) | | 2.7 (0.7) | -13.2 (51.9) | | 2.4 (0.9) | | 2.3 (0.8) | 1.2 (18.1) | | 2.6 (1.1) | 2.7 (0.8) | | -10.8 (34.4) |
| **CMTNS: CMAP** | 2.7 (0.9) | 2.6 (1.0) | 3.1 (19.1) | | 2.7 (0.9) | | 2.6 (0.8) | -4.4 (34.4) | | 2.4 (1.0) | | 2.2 (0.9) | 4.8 (24.1) | | 2.8 (1.0) | 2.6 (0.9) | | 2.6 (28.3) |
| **CMTNS: SNAP** | 4.1 (1.3) | 4.2 (1.2) | -7.7 (29.8) | | 4.0 (1.2) | | 4.0 (1.4) | -1.4 (35.3) | | 3.9 (1.4) | | 4.1 (1.3) | -24.8 (90.4) | | 4.2 (1.1) | 4.1 (1.0) | | -3.1 (29.3) |
| **ONLS** | 3.1 (1.1) | 3.3 (0.9) | -11.8 (33.7) | | 3.3 (1.0) | | 3.6 (0.9) | -12.7 (31.7) | | 3.5 (0.9) | | 3.4 (0.9) | 1.2 (16.7) | | 3.6 (0.8) | 3.4 (1.0) | | 6.8 (18.2) |
| **ONLS: Arm** | 2.4 (0.8) | 2.4 (0.8) | 0.88 (16.2) | | 2.7 (0.7) | | 2.9 (0.7) | -6.4 (16.2) | | 2.7 (0.7) | | 2.7 (0.7) | -1.6 (26.8) | | 2.7 (0.7) | 2.5 (0.8) | | 11 (19.7) |
| **ONLS: Leg** | 2.7 (0.5) | 2.9 (0.3) | -10.5 (20.9) | | 2.6 (0.5) | | 2.8 (0.4) | -7.9 (22.1) | | 2.8 (0.4) | | 2.7 (0.5) | 1.6 (7.3) | | 2.8 (0.4) | 2.9 (0.3) | | -2.6 (11.5) |
| **6MWT (m)** | 468.2 (99.9) | 509.2 (107.7) | 9.0 (8.3) | | 473.1 (70.9) | | 500.9 (75.7) | 6.2 (8.3) | | 450.7 (71.1) | | 481.4 (95.2) | 6.4 (9.4) | | 429.3 (83.7) | 472.0 (98.0) | | 9.9 (6.9) |
| **9HPT (s)** | 17.2 (2.5) | 16.6 (3.0) | 3.6 (10.9) | | 16.1 (3.9) | | 16.3 (3.5) | -2.5 (12) | | 18.4 (4.7) | | 17.4 (3.8) | 4.4 (9.5) | | 20.8 (7.8) | 19.1 (5.9) | | 6.1 (10.6) |
| **Ankle Dorsiflexion (Nm)** | 7.8 (6.6) | 7.4 (5.6) | 20.2 (88.4) | | 9.1 (5.0) | | 7.7 (3.6) | -3.6 (43.0) | | 8.3 (5.6) | | 8.3 (5.1) | 81.5 (369.6) | | 8.2 (6.1) | 7.9 (5.2) | | 20.4 (64.1) |
| **Grip (kg)** | 22.6 (10.7) | 24.5 (12.0) | 9.9 (24.2) | | 21.6 (6.1) | | 22.1 (7.8) | 1.3 (15.6) | | 23.1 (9.2) | | 24.0 (9.3) | 4.7 (12.5) | | 20.6 (10.4) | 22.4 (10.3) | | 11.7 (18.1) |
| **CMAP (milliV)** | 3.7 (2.0) | 4.4 (2.0) | 34.4 (62.0) | | 4.0 (1.8) | | 3.5 (1.7) | 1.4 (38.7) | | 3.7 (2.1) | | 4.0 (2.2) | 22.9 (62.6) | | 3.4 (2.3) | 3.9 (2.1) | | 64.2 (208.5) |
| **MCV (m/s)** | 21.5 (3.6) | 22.4 (4.7) | 3.7 (8.5) | | 22.7 (4.7) | | 22.5 (5.6) | 3.0 (11.5) | | 20.8 (4.8) | | 21.6 (3.8) | 5.7 (12.3) | | 20.5 (5.3) | 21.6 (4.8) | | 9.0 (17.6) |
| **DML (ms)** | 8.6 (2.2) | 8.6 (2.3) | -0.33 (8.7) | | 7.9 (2.1) | | 7.9 (2.0) | 0.33 (16.1) | | 8.2 (1.8) | | 7.3 (1.3) | 8.3 (18.1) | | 8.2 (1.9) | 7.6 (1.4) | | 5 (15.2) |
| **SNAP (microV)** | 2.6 (3.2) | 2.6 (2.9) | 12.4 (121.7) | | 2.3 (3.0) | | 3.0 (3.7) | 11.5 (88.2) | | 2.6 (3.8) | | 2.8 (3.6) | 23.3 (128.4) | | 2.2 (2.7) | 2.5 (2.7) | | 5.2 (69.0) |
| **SCV (m/s)** | 31.1 (14.8) | 31.3 (12.1) | 3.4 (11.0) | | 29.4 (8.2) | | 30.9 (7.5) | 5.3 (11.2) | | 31.3 (9.4) | | 33.9 (9.1) | 29.5 (63.4) | | 29.9 (7.7) | 35.8 (10.4) | | 30.5 (10.0) |
| **VAS: Pain** | 83.9 (23.1) | 80.8 (21.2) | 0.8 (32.0) | | 85.6 (16.8) | | 70.9 (23.7) | -16.7 (23.4) | | 82.0 (17.5) | | 84.3 (16.7) | 9.1 (41.3) | | 80.9 (22.2) | 71.1 (24.0) | | -6.7 (44.3) |
| **VAS: Fatigue** | 61.6 (27.4) | 65.8 (26.3) | 29.8 (80.3) | | 70.0 (20.8) | | 57.1 (26.1) | -17.4 (33.7) | | 70.2 (18.6) | | 61.2 (22.3) | -6.8 (40.5) | | 62.6 (29.6) | 57.3 (26.4) | | -1.0 (39.7) |
| **VAS: Global** | 65.5 (25.3) | 74.7 (18.6) | 51.3 (152.4) | | 72.5 (18.9) | | 71.4 (18.5) | 1.8 (27.8) | | 64.4 (27.5) | | 71.4 (21.2) | 62.3 (161.6) | | 78.4 (16.5) | 70.8 (19.8) | | -8.1 (26.3) |
| **CGI: Global Improvement** | 4.0 (0.0) | 4.1 (0.2) | -1.3 (5.7) | | 4.0 (0.0) | | 4.0 (0.3) | 0 (7.9) | | 4.0 (0.0) | | 3.7 (0.5) | 7.1 (11.6) | | 4.0 (0.0) | 4.1 (0.3) | | -2.6 (7.9) |
| **CGI: Illness Severity** | 3.5 (1.1) | 3.4 (1.1) | 0.61 (22.1) | | 3.5 (0.8) | | 3.5 (0.9) | -2 (30.8) | | 3.5 (0.7) | | 3.7 (1.1) | -8.1 (29.2) | | 3.7 (0.9) | 3.8 (0.9) | | -5.3 (16.7) |
| **CGI: Therapeutic Effect** | 4.0 (0.0) | 3.9 (0.2) | 1.3 (5.7) | | 4.0 (0.0) | | 4.0 (0.2) | 1.2 (5.5) | | 4.0 (0.0) | | 3.7 (0.6) | 8.3 (14.4) | | 4.0 (0.0) | 4.0 (0.0) | | 0 (0) |

|  | **PLI**  (*n* = 61) | | | **PXT3003 LD**  ***versus* Placebo** | | **PXT3003 ID**  ***versus* Placebo** | | **PXT3003 HD**  ***versus* Placebo** | | **PXT3003 HD**  ***versus* PLI** | | **Dose-effect** | |
| --- | --- | --- | --- | --- | --- | --- | --- | --- | --- | --- | --- | --- | --- |
|  | **Baseline** | **Final** | **%**  **Improvement** | **Estimate** | ***P*-value** | **Estimate** | ***P*-value** | **Estimate** | ***P*-value** | **Estimate** | ***P*-value** | **Correlation** | ***P*-value** |
| **CMTNS** | 13.9 (4.0) | 14.0 (3.9) | -3.4 (18.4) | -2.6 (-11.9;7.6) | 0.67 | -3.1 (-11.0;5.4) | 0.74 | 5.5 (-3.4;15.2) | 0.16 | 8.0 (0.4;16.2) | 0.042 | 0.06 | 0.3 |
| **CMTES** | 9.3 (3.1) | 9.5 (3.0) | -5.2 (23) | -4.9 (-15.4;6.9) | 0.76 | -1.9 (-12.1;9.4) | 0.62 | 6.3 (-5.3;19.2) | 0.19 | 8.7 (-0.39;18.6) | 0.058 | 0.034 | 0.38 |
| **CMTNS: Sensory Symptoms** | 1.8 (1.0) | 1.8 (1.0) | -8.7 (49.9) | -13.0 (-31.8;10.9) | 0.83 | -6.6 (-23.7;14.2) | 0.72 | 14.0 (-8.1;41.3) | 0.16 | 21.0 (1.4;44.3) | 0.038 | 0.081 | 0.24 |
| **CMTNS: Motor Symptoms: Legs** | 2.3 (0.7) | 2.2 (0.8) | 3.3 (19.4) | -2.6 (-11.6;7.3) | 0.68 | 7.4 (-5.5;22.1) | 0.18 | -3.3 (-8.0;1.7) | 0.87 | -5.3 (-13.4;3.6) | 0.84 | -0.038 | 0.63 |
| **CMTNS: Motor Symptoms: Arms** | 1.8 (0.6) | 1.7 (0.6) | 0.6 (27.2) | -5.1 (-15.1;6.2) | 0.78 | -0.1 (-12.6;14.2) | 0.51 | -0.007 (-9.5;10.5) | 0.5 | 1.1 (-8.2;11.4) | 0.42 | -0.02 | 0.57 |
| **CMTNS: Pin Sensitivity** | 2.4 (1.0) | 2.4 (0.9) | -8.7 (46.6) | 18.1 (-3.9;45.2) | 0.091 | -6.4 (-20.6;10.4) | 0.75 | 15.3 (-6.3;42.0) | 0.13 | 9.4 (-7.5;29.4) | 0.19 | -0.056 | 0.69 |
| **CMTNS: Vibration Sensitivity** | 2.8 (1.0) | 3.3 (1.2) | -26.5 (50.7) | -5.1 (-20.6;13.5) | 0.69 | -3.8 (-19.4;14.8) | 0.64 | 4.8 (-12.2;25.1) | 0.33 | 8.6 (-5.3;24.4) | 0.16 | 0.017 | 0.44 |
| **CMTNS: Strength: Legs** | 2.6 (0.8) | 2.6 (0.7) | -1.2 (18.3) | -3.7 (-12.8;6.2) | 0.74 | -2.8 (-10.1;5.2) | 0.73 | 3.6 (-5.0;13.0) | 0.25 | 6.1 (-1.2;13.9) | 0.084 | 0.085 | 0.23 |
| **CMTNS: Strength: Arms** | 2.5 (0.8) | 2.5 (0.7) | -5.5 (34.6) | -2.4 (-14.2;11.0) | 0.63 | 7.1 (-3.0;18.2) | 0.12 | -3.6 (-12.5;6.2) | 0.73 | -5.5 (-14.0;3.8) | 0.84 | 0.012 | 0.46 |
| **CMTNS: CMAP** | 2.6 (0.9) | 2.5 (0.9) | 1.1 (26.7) | -3.5 (-16.4;11.4) | 0.66 | 4.8 (-7.9;19.1) | 0.27 | 0.2 (-11.1;13.0) | 0.49 | -0.8 (-11.0;10.7) | 0.55 | 0.089 | 0.22 |
| **CMTNS: SNAP** | 4.0 (1.3) | 4.1 (1.3) | -11.4 (59.2) | 9.9 (-7.1;30.0) | 0.17 | -1.4 (-16.7;16.7) | 0.56 | 3.2 (-8.3;16.1) | 0.33 | 0.7 (-12.5;15.9) | 0.47 | 0.024 | 0.42 |
| **ONLS** | 3.3 (1.0) | 3.4 (0.9) | -7.7 (28.5) | -3.9 (-14.2;7.6) | 0.72 | 6.9 (-3.8;18.8) | 0.15 | 14.4 (0.55;30.2) | 0.043 | 12.1 (2.0;23.2) | 0.024 | 0.28 | 0.0059 |
| **ONLS: Arm** | 2.6 (0.8) | 2.6 (0.8) | -2.5 (20.4) | -8.1 (-15.2;-0.41) | 0.96 | -2.8 (-14.2;10.2) | 0.65 | 13.7 (0.22;29.0) | 0.047 | 15.7 (4.9;27.6) | 0.0076 | 0.22 | 0.027 |
| **ONLS: Leg** | 2.7 (0.5) | 2.8 (0.4) | -5.5 (18.4) | 4.5 (-3.0;12.5) | 0.16 | 9.7 (3.1;16.7) | 0.0078 | 3.0 (-2.7;9.0) | 0.19 | -0.9 (-6.2;4.7) | 0.61 | 0.21 | 0.03 |
| **6MWT (m)** | 463.8 (80.0) | 496.6 (92.1) | 7.1 (8.6) | -2.4 (-6.2;1.5) | 0.85 | -2.4 (-6.6;2.0) | 0.82 | 0.7 (-3.2;4.7) | 0.38 | 2.6 (-0.73;6.1) | 0.099 | 0.11 | 0.16 |
| **9HPT (s)** | 17.2 (3.9) | 16.8 (3.4) | 1.8 (11.1) | -4.6 (-10.3;1.5) | 0.89 | -0.2 (-5.3;5.2) | 0.52 | 0.3 (-5.7;6.6) | 0.47 | 1.2 (-3.4;6.0) | 0.33 | 0.15 | 0.092 |
| **Ankle Dorsiflexion (Nm)** | 8.4 (5.7) | 7.8 (4.7) | 33.1 (223.2) | -4.0 (-21.7;17.8) | 0.63 | 11.4 (-15.4;46.8) | 0.26 | 8.2 (-13.8;35.9) | 0.28 | 5.5 (-12.8;27.7) | 0.32 | 0.11 | 0.16 |
| **Grip (kg)** | 22.4 (8.7) | 23.5 (9.7) | 5.1 (17.9) | -7.1 (-15.6;2.1) | 0.9 | -3.6 (-11.8;5.4) | 0.75 | 1.6 (-7.7;11.9) | 0.39 | 6.0 (-1.2;13.7) | 0.088 | 0.12 | 0.15 |
| **CMAP (milliV)** | 3.8 (2.0) | 4.0 (2.0) | 19.6 (56.5) | -25.1 (-44.8;1.5) | 0.94 | -9.2 (-27.3;13.5) | 0.77 | -5.1 (-27.1;23.6) | 0.63 | 6.6 (-15.8;35.1) | 0.33 | -0.0011 | 0.5 |
| **MCV (m/s)** | 21.6 (4.4) | 22.2 (4.7) | 4.2 (10.9) | -1.0 (-6.5;4.9) | 0.61 | 0.5 (-4.8;6.2) | 0.44 | 2.8 (-3.4;9.4) | 0.23 | 2.5 (-2.4;7.7) | 0.21 | 0.11 | 0.18 |
| **DML (ms)** | 8.2 (2.0) | 7.9 (2.0) | 3 (15.3) | 3.4 (-4.3;11.7) | 0.24 | 13.8 (4.2;24.3) | 0.0092 | 8.0 (0.59;16.0) | 0.038 | 2.2 (-5.1;10.0) | 0.31 | 0.21 | 0.035 |
| **SNAP (microV)** | 2.5 (3.3) | 2.8 (3.4) | 15.9 (110.2) | -1.2 (-42.9;71.0) | 0.52 | 8.7 (-31.2;71.6) | 0.38 | 13.9 (-24.1;71.0) | 0.29 | 12.0 (-23.9;64.9) | 0.31 | 0.089 | 0.3 |
| **SCV (m/s)** | 30.6 (10.7) | 32.0 (9.2) | 12.7 (38.0) | 1.5 (-5.8;9.4) | 0.36 | 17.5 (-5.5;46.2) | 0.11 | 26.6 (15.5;38.8) | 0.00037 | 20.1 (2.4;40.8) | 0.03 | 0.42 | 0.0098 |
| **VAS: Pain** | 83.9 (18.9) | 78.6 (21.2) | -2.4 (34.4) | -17.4 (-30.7;-1.6) | 0.96 | 6.7 (-7.2;22.6) | 0.22 | -13.6 (-29.0;5.3) | 0.89 | -10.3 (-23.2;4.8) | 0.88 | -0.057 | 0.69 |
| **VAS: Fatigue** | 67.5 (22.4) | 61.2 (24.7) | 1.0 (57.1) | -25.6 (-43.1;-2.7) | 0.96 | -10.5 (-28.8;12.6) | 0.79 | -17.0 (-34.4;5.0) | 0.91 | -2.5 (-20.3;19.3) | 0.58 | -0.094 | 0.8 |
| **VAS: Global** | 67.5 (24.0) | 72.4 (19.2) | 38.0 (129.0) | -8.1 (-21.0;6.9) | 0.82 | -11.4 (-33.5;18.0) | 0.76 | -10.5 (-24.5;6.1) | 0.86 | -2.6 (-19.4;17.7) | 0.59 | -0.21 | 0.97 |
| **CGI: Global Improvement** | 4.0 (0.0) | 3.9 (0.4) | 2.1 (9.5) | 1.5 (-2.2;5.3) | 0.25 | 9.8 (4.0;16.0) | 0.0032 | -1.2 (-4.4;2.2) | 0.72 | -4.8 (-8.7;-0.69) | 0.97 | 0.051 | 0.33 |
| **CGI: Illness Severity** | 3.5 (0.8) | 3.5 (1.0) | -3.3 (27.6) | -1.4 (-15.6;15.2) | 0.56 | -8.1 (-18.9;4.1) | 0.87 | -8.3 (-17.5;1.9) | 0.91 | -6.1 (-16.0;5.0) | 0.82 | -0.15 | 0.91 |
| **CGI: Therapeutic Effect** | 4.0 (0.0) | 3.9 (0.4) | 3.7 (10) | -0.1 (-3.5;3.3) | 0.53 | 9.0 (1.1;17.5) | 0.03 | -1.5 (-4.0;1.0) | 0.84 | -4.3 (-8.8;0.36) | 0.94 | 0.042 | 0.35 |
